# Effects of biocontrol *Bacillus* sp. strain D5 on the pathogenic *Fusarium oxysporum* R1 at the microscopic and molecular level in *Crocus sativus* L. (saffron) corm

**DOI:** 10.1093/femsmc/xtad025

**Published:** 2024-01-03

**Authors:** Nancy Bhagat, Jyoti Vakhlu

**Affiliations:** Metagenomics Laboratory, School of Biotechnology, University of Jammu, Jammu, 180006, Jammu and Kashmir, India; Metagenomics Laboratory, School of Biotechnology, University of Jammu, Jammu, 180006, Jammu and Kashmir, India

**Keywords:** corm rot, *Fusarium oxysporum* R1, *Bacillus* sp. strain D5, biocontrol, transcriptome, saffron

## Abstract

Corm rot of saffron caused by *Fusarium oxysporum* is a major threat to saffron cultivation the world over. To minimize the ill effects of chemical fungicides, attention has been shifted to the use of biocontrol agents for disease management in a sustainable way. In saffron, various biocontrol agents against corm rot disease have been reported and characterized but no study has been done so far to understand their interaction at the molecular level. The present study was conducted to unravel the mechanism of action of an already characterized native biocontrol agent i.e. *Bacillus* sp. strain D5 (Bar D5) against *F. oxsporum* R1 (Fox R1) in the saffron corm. The growth inhibition of Fox R1 was observed *in vitro* and *in planta* (saffron corm) by real time imaging. *Bacillus* sp. strain D5 reduced Fox R1 load in infected corms by 50% as quantified by q-PCR and the colony-forming unit method. Comparative transcriptome analysis revealed upregulation and downregulation of various Fox R1 genes in presence of Bar D5. The genes related to carbon metabolism, cell wall and membrane synthesis, and growth of Fox R1 were significantly downregulated in Bar D5-primed and Fox R1-inoculated corms as compared to only Fox R1-inoculated corms.

## Teaser

Bar D5, an efficient biocontrol agent against *Fusarium oxysporum* causing corm rot in saffron.

## Introduction

Saffron (*Crocus sativus* L.) is an economically important crop that produces the costliest spice in the world (Mansotra et al. 2023, Spinelli et al. 2023). Due to the presence of active compounds such as crocin, picrocrocin, safranal, and others in the stigma of saffron, it is widely used as a spice and has extensive application in the pharmaceuticals, cosmetics, and food industries (Kothari et al. 2021, Ali et al. 2023). Recently, it has also been demonstrated to have anti-inflammatory and antiviral potential against the COVID-19 virus (Mentis et al. 2021). Other parts of the saffron plant, such as leaves, tunics, spaths, tepals, and petals have also been used in pharmaceuticals (Naim et al. 2022a,b, Lahmass et al. 2017). Saffron production in Jammu and Kashmir, India is on the constant decline. In 1997, total production was 15 tonnes, which was reduced to 9 tonnes in 2015, and further reduced to 3.8 tonnes in 2021 (Ganaie and Singh 2019, Kumar et al. 2022). The major reason for this decline is the unavailability of healthy cultivable corms that are free from wound or injury, as saffron is vegetatively propagated through corms (Gupta et al. 2021).

Corm rot of saffron caused by pathogenic *Fusarium oxysporum* is the major disease that affects saffron production (Magotra et al. 2021, Bhagat et al. 2022). During corm rot disease progression, the pathogen enters the corm through injuries or wounds, multiplies inside the corm, and moves to the fibrous roots, ultimately leading to plant death (Bhagat et al. 2022, Mansotra et al. 2023). At present, chemical measures are used as a preventative strategy for the management of disease due to their easy application and broad spectrum (Gupta et al. 2020). The ill effects of these chemicals on the environment as well as humans are well documented (Magotra et al. 2021). The excessive use of these chemicals also affects the native microflora, which are important for maintaining the fertility and health of the soil (Pahalvi et al. 2021).

The use of native biocontrol agents has become a promising strategy for the control of soil borne diseases and is an ecofriendly approach (Ram et al. 2018). Among different bacterial genera, *Bacillus* has gained much importance as a potential biocontrol agent due to the formation of heat and desiccation-resistant spores (Kavitha et al. 2023, Singh et al. 2023). Various *Bacillus* strains have been isolated and characterized from saffron plants and fields by our group as plant growth promoting bacteria and biocontrol agents against Fox R1, such as *Bacillus amyloliquefaciens* W2 (Gupta and Vakhlu 2015), a consortium of three bacilli, i.e. *Bacillus thuringiensis* DC1, *Bacillus megaterium* VC3, and *B. amyloliquefaciens* DC8 (Kour et al. 2018) and *Bacillus* sp. strain D5 (Magotra et al. 2021). Among these, *Bacillus* sp. strain D5 (Bar D5) isolated from the saffron cormosphere was found to be the best, in terms of saffron growth promotion and biocontrol activity against Fox R1 in pots as well as in fields (Magotra et al. 2021). Moreover, the draft genome sequence of Bar D5 revealed the presence of genes related to stress tolerance and production of antifungal VOCs (Magotra et al. 2021).

The mechanism of antagonism to *F. oxysporum* by any *Bacillus* strain or any other biocontrol agent has not been deciphered so far in saffron. In the present study, the antagonist mechanism of action of the Bar D5 antagonist to Fox R1 was studied *in planta* in real time. Further, the analysis of differentially expressed genes (DEGs) of Fox R1 in the presence of Bar D5 revealed the molecular mechanism of decreased infectivity of Fox R1 toward the saffron plant. Further, Bar D5 was demonstrated to have a long-term inhibition against Fox R1 in comparison to the commonly used chemical fungicide, carbendazim; therefore, Bar D5 can be used to replace these chemical agents in the future.

## Materials and methods

### Corm sample, bacterial and fungal strains, and culture conditions

Saffron corms were procured from the Pampore region (34.0060°N, 74.9238°E) of the Pulwama district of the Kashmir region, Jammu and Kashmir, during its dormant phase in August 2020.

Already isolated wild type pathogenic *F. oxysporum* R1 (GenBank accession number of the ITS gene: *KF663598*) causing corm rot in saffron and EGFP-tagged *F. oxysporum* R1 was used in the present study (Gupta and Vakhlu 2015, Bhagat et al. 2022). The wild type Fox R1 was cultivated on potato dextrose agar (PDA) plates (Difco, BD Becton, and Dickinson) and EGFP-tagged Fox R1 was cultivated on PDA plates supplemented with 100 µg/ml of hygromycin-B and incubated at 25°C for 5 days in the Orbitek incubator (Scigenics Biotech, India). *Bacillus* sp. strain D5 (GenBank accession number of the 16S rRNA gene: *KT228251*) was previously isolated from the cormosphere (Magotra et al. 2021). Bar D5 was cultured on Nutrient Agar (NA; Himedia) plates and incubated at 37°C for 24 hours in an Orbitek incubator (Scigenics Biotech).

### *In vitro* antagonism assays of Bar D5 against *F. oxysporum* R1

#### Dual culture method

The antifungal activity of Bar D5 was tested by dual culture method (Ji et al. 2013). In brief, 5 mm agar plug of Fox R1 from a 7-day-old culture was taken and placed on one edge of the Muller Hinton agar plates and on another edge, overnight grown Bar D5 culture was streaked at a distance of 5 cm. The control plates were without Bar D5 culture. The plates were incubated at 28 ± 1°C for 5 days. The photographs of the plates were subjected to ImageJ software for measuring the diameter of Fox R1 in both control and test plates (Abramoff et al. 2004). The inhibition percentage was calculated using the formula:


\begin{eqnarray*}
&& {\rm Inhibition\,\, percentage} = ({\rm Control-test})/{\rm Control} \times 100\\
&& {\rm Control} = {\rm diameter\,\, of\,\, growth\,\, of\,\, the\,\, Fox\,\, R1\,\, in\,\, the\,\, control\,\, plate,}\\
&& {\rm and}\\
&& {\rm Test} = {\rm growth\,\, of\,\, the\,\, Fox\,\, R1\,\, in\,\, the\,\, test\,\, plate}.
\end{eqnarray*}

#### Effect of the cell-free extract of Bar D5 on morphology and growth of Fox R1

The effect of Bar D5 cell-free extract was studied on Fox R1 morphology by visualizing the Fox R1 hyphae under the light microscope (Gupta and Vakhlu 2015). Bar D5 was grown overnight in nutrient broth (NB; Himedia) and cell-free extract was collected by centrifuging the broth at 10,000 *g* for 10 minutes at 4°C. A volume of 2 ml of cell-free extract was mixed with 8 ml of PDB, and Fox R1 spores were inoculated in it. For control, instead of cell-free extract 2 ml of sterile distilled water was added to 8 ml of PDB. Both test tubes were incubated at 28°C in the Orbitek incubator shaker (Scigenics Biotech) for 10 days at 180 rpm. The morphology of Fox R1 in control and test was observed under the light microscope using lactophenol cotton blue dye after 10 days of incubation.

#### Effect of VOCs of Bar D5 on morphology and growth of Fox R1

The production of VOCs by Bar D5 and its antifungal potential was determined by the seal plate method (Fernando et al. 2005). The Bar D5 was streaked on the bottom dish of the NA plate and incubated at 37°C overnight in an Orbitek incubator (Scigenics Biotech). The 5 mm plug of Fox R1 was placed at the center of the bottom of the PDA dish and inverted over the NA plate containing Bar D5 culture; both dishes were sealed together with 3 layers of parafilm. The control plates were inoculated with Fox R1 and other plate was uninoculated. The plates were incubated at 28°C for 7 days in the Orbitek incubator (Scigenics Biotech). After 7 days, the diameter of growth of Fox R1 in the control and test plates was determined using ImageJ software (Abramoff et al. 2004). The growth inhibition was calculated by using the formula mentioned in the section “Dual culture method.” Further, the Fox R1 hyphae from both the plates were stained with lactophenol cotton blue dye and visualized under the light microscope. Three replicates for all the *in vitro* experiments were taken and each experiment was performed thrice at different time intervals.

### *In planta* antagonism assays of Bar D5 against Fox R1

The *in planta* effect of Bar D5 against Fox R1 was evaluated in pot trials. The bioformulation of Bar D5 (10^8^ CFU/ml) was prepared following the standardized protocol of Magotra et al. (2021). In brief, Bar D5 was grown in NB for 48 hours and a colony count of 10^8^ CFU/ml was maintained. The broth was mixed with double autoclaved calcium carbonate (talc) in a ratio of 1:2 (v/w) and dried for 3–4 days at 35–37°C. Finally, 1% sterile carboxymethyl cellulose was added to the dried bioformulation. For the priming of saffron corms, slurry of bioformulation was prepared by mixing a 100 gm of dried bioformulation with 100 ml of sterile distilled water.

A total of 72 saffron corms ranging between 8 and 10 gm were taken and divided into two sets: control, mock primed and test, Bar D5 primed. A total of 24 corms were primed with Bar D5 bioformulation, and remaining 48 corms were mock primed with calcium carbonate only (Magotra et al. 2021). Both sets of corms were dried overnight at room temperature and then sown in an autoclaved sand and soil mixture (1:1 W/W) in pots of 76 mm mouth diameter with 1 corm/pot. The pots were incubated in a plant growth chamber at 25 ± 2°C under a 16-hour light/8-hour dark cycle for 30 days. The spore suspension of EGFP-labeled Fox R1 (10^12^ spores/ml) was prepared as per the standardized protocol of our group (Bhagat et al. 2022). After 30 days, corms were taken out and an artificial injury was given to each corm with the help of a sterile needle (2 cm × 1 cm) and placed back into their respective pots. The 1 ml EGFP-labeled Fox R1 spore suspension (10^12^ spores/ml) was added to the pot soil and the pots were again incubated at the same conditions as mentioned above. Further, the corms were taken out at 1, 2, 3, 4, 5, 8, 12, and 20 days postinoculation (dpi) for real time imaging and load quantification of Fox R1 by real time PCR and the colony-forming unit (CFU) method. The experiment layout has been tabulated in Table 1.

**Table 1.** Experiment layout for the quantification of Fox R1 load in different treatments using q-PCR and CFU method.

		Procedure					
S.No	Treatments	Step-1 Corm priming	Step-2 Incubation	Step-3 Injury	Step-4 Inoculation	Step-5 Incubation	No. of corms
1	Pathogen (Mock-primed and Fox R1-inoculated corms)	Mock primed	Sowing of corms in pots and incubated for 30 days at 25 ± 2°C under 16 hours light/8 hours dark cycle	+	1 ml of EGFP-labeled Fox R1 spore suspension (10^12^ spores/ml) was added to pot soil	Corms were replanted and incubated at 25 ± 2°C under 16 hours light/8 hours dark cycle	24
2	Test 1* (Carbendazim-treated and Fox R1-inoculated corms)	Mock primed		+			24
3	Test 2 (Bar D5-primed and Fox R1-inoculated corms)	Bar D5 primed		+			24

* In test 1 before Fox R1 spore suspension inoculation, corms were treated with chemical fungicide carbendazim at a concentration of 100 µg/ml by dipping the corms in fungicide solution for 30 minutes.

#### Real time imaging of EGFP-labeled Fox R1 in infected saffron corms with different treatments using confocal microscope

To visualize the effect of Bar D5 on EGFP-labeled Fox R1 inside the saffron corm tissue, the corm samples at 1, 2, 3, 4, 5, 8, 12, and 20 dpi were taken out from their respective pots and washed gently with sterile distilled water to remove the soil particles adhered to the corm surface and air dried for 30 minutes. The corms were dissected into two halves and observed for visual symptoms. Further, the infected part of the corm tissues was excised manually, cut into thin sections with the help of a sterile blade, and placed on the glass slide in a drop of sterile water (Bhagat et al. 2022). The tissue section was covered with a cover slip and directly observed under the confocal microscope at 60X magnification. The tissue was scanned in multiple planes (Z stack) under a confocal microscope (Leica TCS SP8 AOBS, NIPGR, New Dehli).

#### Quantification of EGFP-labeled Fox R1 in infected saffron corms with different treatments by using CFU method

To quantify the EGFP Fox R1 in infected corm tissue with the different treatments mentioned in Table 1, Komada medium was used (Komada 1975). As EGFP-labeled Fox R1 is resistant to hygromycin, the medium was supplemented with 100 µg/ml of hygromycin-B antibiotic that acted as a selectable marker for the growth of EGFP-labeled Fox R1 as wild-type Fox R1 is susceptible to this concentration of hygromycin. The infected tissues from different treatments were taken and washed with sterile distilled water under sterile conditions, inside laminar airflow. A total of 100 mg of infected tissue was crushed using a sterile pestle and mortar and then 1 ml of autoclaved distilled water was added. Serial dilution of the suspension was done, and 10^−2^ dilution was spread on the Komada media plates and incubated at 28°C for 5 days in an Orbitek incubator (Scigenics Biotech). After 5 days, colonies were counted and the load was determined as the number of Fox R1 colonies per gram of corm tissue in different treatments. The inhibition percentage was calculated using the below mentioned formula.


\begin{eqnarray*}
&& {\rm Inhibition\,\, percentage}\\
&&\quad = \left({\rm Fox\,\, R1\,\, colonies\,\, in\,\, the\,\, test\,\, plate}/{\rm Fox\,\, R1\,\, colonies\,\, in\,\,} \right.\\
&&\quad\quad \left. {\rm the\,\, control\,\, plate} \right ) \times 100.
\end{eqnarray*}

#### Quantification of EGFP-labeled Fox R1 in infected saffron corms with different treatments using q-PCR

The abundance of EGFP-labeled Fox R1 in different treatments (Table 1) was quantified by q-PCR using the ITS gene, which has already been characterized for its selectivity and sensitivity in our previous study (Bhagat et al. 2022). The *crocus* 18S rRNA gene was used as the reference gene (RG) for the normalization of data (Table 2). The genomic DNA was extracted from infected tissues to obtain DNA of both Fox R1 and corm from different treatments, as shown in Table 1 at 1, 3, 5, 8, 12, and 20 dpi using the CTAB method (Doyle and Doyle 1990). Relative quantification of Fox R1 was done in carbendazim-treated and Fox R1-inoculated corms (Test 1) and Bar D5-primed and Fox R1-inoculated corms (Test 2) compared to Fox R1-inoculated corms (control) at 1, 3, 5, 8, 12, and 20 dpi using a SYBR green dye-based assay. The PCR reaction mixture consisted of SYBR green master mix—5 µl (Thermo-Scientific), forward primer—1 µl, reverse primer—1 µl, DNA template—1 µl, and MiliQ—2 µl for total reaction volume of 10 µl and the reaction was performed in 8-well strips on a 7500 Real Time PCR System (Applied Biosystems®). The PCR program used was initial denaturation at 95°C for 10 minutes, followed by 40 cycles of denaturation at 95°C for 15 seconds, annealing at 60°C for 1 minute, and extension at 72°C for 30 seconds. The relative quantification was performed using the formula:


\begin{eqnarray*}
&& {\rm Relative\,\, quantification} = {2}^{- (\Delta {\rm Ct test} - \Delta {\rm Ct control})}\\
&&\quad\quad\quad\quad {\Delta {\mathrm{Ct test}} = {\mathrm{Ct TG}} - {\mathrm{Ct RG}}}\\
&&\quad\quad\quad\quad {\Delta {\mathrm{Ct control}} = {\mathrm{Ct TG}} - {\mathrm{Ct RG}}},
\end{eqnarray*}

where TG—test gene, RG—reference gene, Control = corm tissue inoculated with Fox R1, Test1—carbendazime-treated and Fox R1-inoculated saffron corm, Test2—Bar D5-primed and Fox R1-inoculated saffron corm.

**Table 2.** Prime pair used for the *in planta* quantification of EGFP-labeled *F. oxysporum* R1.

S. No	Target gene	Primer	Primer Sequence (5’–3’)	Amplicon size (bp)
1	Fox R1 ITS gene	Forward	GAACGCACATTGCGCCCGCCAGTA	100
		Reverse	CGCGAATTAACGCGAGTCCCAACA	
2	*Crocus* 18S gene	Forward	CGGCGCAGTGGGCGCCAAGG	123
		Reverse	CGCCTGCGTCCCTCCTCCTCCCT	

### Transcriptome analysis of *F. oxysporum* R1 in saffron corm

The total RNA was isolated from control, i.e. mock-primed corms and inoculated with Fox R1 spore suspension and test, i.e. Bar D5-primed and Fox R1-inoculated corms at 2 dpi using Trizol regent. The quality and integrity of the RNA was checked by agarose gel electrophoresis on 1.2% agarose gel. The integrity of the RNA was determined by the 260/280 absorption ratio by nanodrop. Further, the RNA Integrity Number was determined using an Agilent 2100 Bioanalyzer (Agilent, USA). cDNA libraries were prepared using the True-Seq^R^ RNA sample preparation kit v2 (Illumina CA, USA) following the manufacturer’s instructions, and the sequencing was done using the Illumina Hiseq 2500 system (2 bp × 150 bp) with default parameters. The raw data obtained after sequencing was processed as follows: (1) FastQC of the raw reads was performed using the FastQC v0.11.5 tool (Andrews 2017). The good-quality reads with a phred score Q > 30 were selected and further processed for alignment and assembly; (2) the reference genome of *F. oxysporum* f.sp. *lycopersici* 4287 (http://fungi.ense mbl.org/Fusarium_oxysporum/Info/Index) was downloaded from NCBI and raw reads were aligned to it using the software Hisat2 v2.1.0 (Kim et al. 2015); (3) the aligned reads were further processed for reference-guided transcript assembly and for quantification of the transcripts using the software Stringtie v1.3.5 (Pertea et al. 2015). The number of fragments per kilobase of transcript sequence per million base pairs sequenced (FPKM) was used to estimate the expression level of each gene in all the samples; (4) the DEGs were analyzed using the Bioconductor package DESeq2 v1.26.1 in the R environment (Varet et al. 2016); and (5) for functional annotation of the DEGs, Gene Ontology (GO) and Kyoto Encyclopaedia of Genes and Genomes (KEGG) was done using the g: Profiler web server (Raudvere et al. 2019). The putative function of all the genes was determined using the Biomart tool of ensemble fungi (Kinsella et al. 2011).

### Validation of selected DEGs with quantitative reverse transcription PCR (RT-qPCR)

The gene sequences of 20 genes from the transcriptome data were selected and used for primer design. The primers were designed using the PrimerQuest tool of Integrated DNA Technologies (http://www.idtdna.com/SciTools/SciTools.aspx). The primer set design has been mentioned in the Table 3. Total RNA was extracted and. cDNA was synthesized from 10 µg of the total RNA using high-capacity cDNA reverse transcription kit (Applied Biosystems, catalog number 4368814) following the manufacturer’s protocol. The q-PCR reaction mixture (10 µl) consisted of SYBR green master mix (5 µl), cDNA template (1 µl), and gene specific primers (0.5 µM each). The q-PCR was performed in 8-well strips using SYBR green-based assay (Thermo-Scientific, catalog number 4309155) on 7500 real time PCR System (Applied Biosystems® model). The relative quantification was done by 2^−ΔΔCT^ method (Fleige and Pfaffl 2006). For the quantification of Fox R1 genes, its actin gene was used as reference gene for the normalization of gene expression.

**Table 3.** List of saffron *F. oxysporum* R1 genes validated by RT-qPCR along with primer sequence.

Gene ID	Gene name	Category	Forward (5'–3')	Reverse (5'–3')	Amplicon size (bp)
FOXG_12330	*pme*	Plant cell wall degrading enzymes	TCGCCAACGGTGCTTAC	CCCTTGGAGTTGATGACAGAG	97
FOXG_08862	*pgx1*		GTTGGGTCTCGGCATGTAATA	CGTGGCTGAGGATATCAAAGT	105
FOXG_19077	*pl1*		GCTCTCCAATCGGTCTCTTTC	GATGTCTGGCCGCTATTGT	107
FOXG_15742	*xyl4*		GCCGTCAACATGCAGACTA	GAAGATCCACTGCTCTGGTAAC	110
FOXG_13051	*pg5*		CGGTTCCGTTTCCGATGT	CCGTTCTCGTAATCCTGCTC	90
FOXG_06378	*fow2*	Transcription factors	CGTGCTTCTATCTCAAGAAGGG	CCCTGATGTTCCTGTCAATGT	99
FOXG_03748	*xnlr*		GGCGTGGTATTCTTCCTTCTG	GGGTGGGATTGAGACATTCG	100
FOXG_10510	*sge1*		TGTGTGTGGGAAAGCCAATA	CCGATCGAGGAAGCTCAAC	94
FOXG_02103	*ste12*		GGCGGAGATGGTTCCTTG	GAGTGTGGTGAGGCTTCTTC	95
FOXG_08140	*fmk1*		GGGTTGTGCTGTGGTCTATC	AGATCGGCGAGGCATCTA	143
FOXG_03165	*fnr1*		GTCCTGTCCCATCCATCAAAT	GATTGGCCGAGGGTTTCA	101
FOXG_00058	*frp1*	F-box protein	TCGAGCGTCCACAGGATA	GTACCACCATTCGTCCTCTTC	92
FOXG_04162	*chsv*	Chitin synthase	GATCCCACCCATCTACCAAAC	GGTAGTTAGCGAGGGAGAGTAT	104
FOXG_03165	*fdp1*	Ion conductance	GTCCTGTCCCATCCATCAAAT	GATTGGCCGAGGGTTTCA	101
FOXG_09359	*fga1*	G-protein	CTCACCCATTACGCCAGATATAC	GGCAACTCCCATCCATCAA	102
FOXG_11292	*fow1*	Mitochondrial carrier protein	TGTGCTGGGACTGGTTTAC	TGGATCTGACTCAGCATTTAGG	117
FOXG_01957	*arg1*	Argionsuccinate lyase	GCTGGATTCGGTCCAAACT	AGTGGCAAGCATCGTCTTATC	118
FOXG_01489	*cnb1*	*Calcineurin* subunit B	TGTGTAGATTCGCTAGGTGTTC	GCATACAGGGTAGGAGAGTAAAG	125
FOXG_10292	*adh1*	Alcohol dehydrogenase	CCCTTGGTCGTGGTATCTTG	TGAAGTTGTCACCCTGGAAC	102
FOXG_03721	*fks1*	1,3-beta-glucan synthase	GGAGAGCGTAGGCTAGTAGTATAA	GAAACACATCCATGGCAGAAAC	95
FOXG_05015	*act*	Reference gene	CGAGATGACCTTGACCTTTGT	GGCTTCTGCATCGCTCTATC	94

### Statistical analysis

Results were expressed as the mean ± standard deviation. The data was statistically analyzed by ANOVA using IBM SPSS Statistics version 26. The Multiple Duncan range test was performed to analyze differences between mean values at significant level (*P* < .05). All the experiments were replicated independently three times at three different time intervals.

## Results

### *In vitro* antagonism of Bar D5 against Fox R1

**Figure 1.**
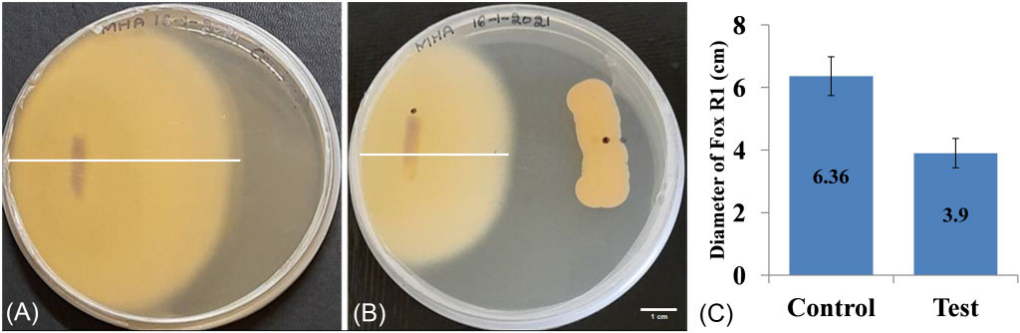
Antagonism of *Bacillus* sp. strain D5 (Bar D5) by dual culture against *F. oxysporum* R1 (Fox R1) (A) Control (Fox R1 only). (B) Test (Fox R1 + Bar D5) Bar represents 1 cm. (C) Bar plot representing the diameter of Fox R1 growth in control and test. Error bar represents the standard deviation.

**Figure 2.**
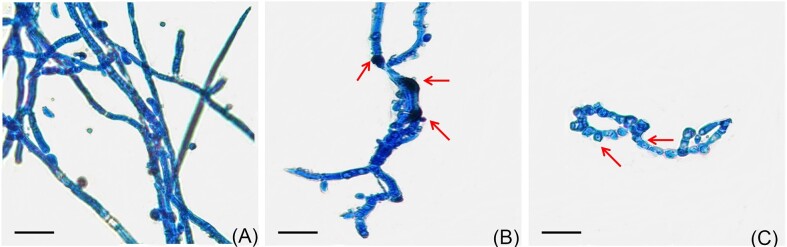
Effect of cell-free extract of Bar D5 on Fox R1 morphology after 10 days of incubation. (A) Control; Fox R1 grown in PDB, (B) and (C), Test; Fox R1 grown in the cell-free extract of Bar D5. Arrows in (B) represents the thickening of cell wall whereas in (C) represents shortening of hyphae. Each bar represents 50 µm.

In the dual culture assay, the diameter of growth of Fox R1 in control and test was found to be 6.36 ± 0.62 cm and 3.9 ± 0.47cm, respectively and the inhibition percentage was found to be 38.6% (*P* = .00) in the presence of Bar D5 (Fig. 1). The incubation of Fox R1 spores in the cell-free extract of Bar D5 (test) resulted in structural deformities in hyphae such as thickening of cell wall (Fig. 2B), changes in the morphology, and shortening of hyphae (Fig. 2C). The hyphae were shrunken and round in structure (Fig. 2C). However, in the control, *F. oxysporum* R1 hyphae were growing normally, elongated, visible with numerous spores, and the formation of a germ tube from the hyphae was also observed (Fig. 2A).

**Figure 3.**
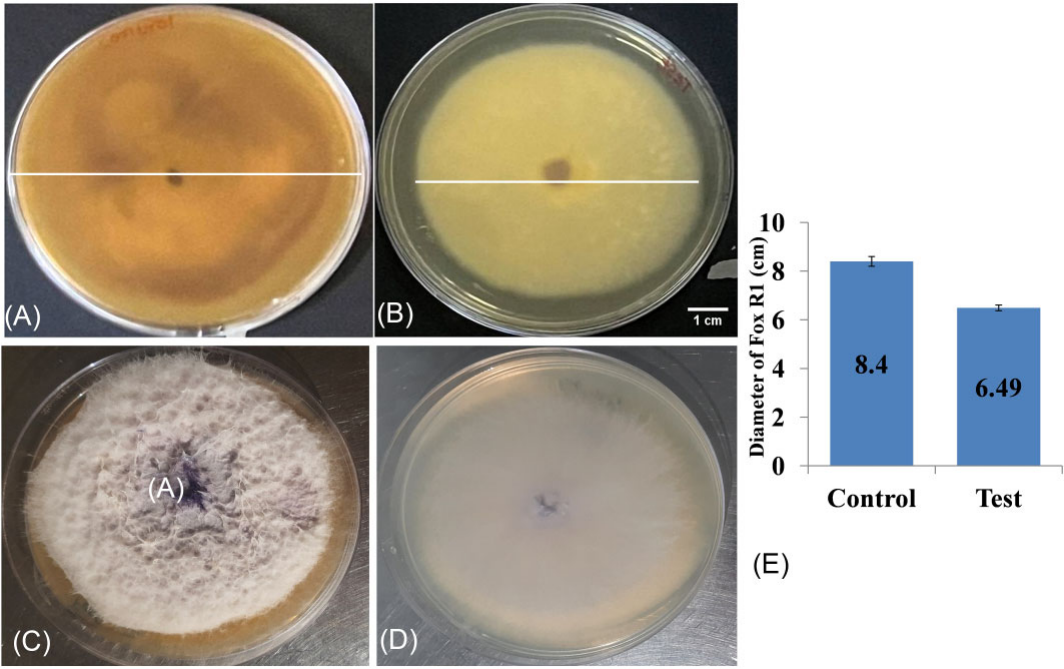
Effect of *Bacillus* sp. strain D5 volatile compound on *F. oxysporum* R1 (Fox R1) by sealed plate method. (A) and (C) Control (B) and (D) Test (Fox R1 grown in presence of volatile compound), after 7 days of incubation, complete loss of pigmentation was observed. Bar represents 1 cm. (E) Bar plot represents the diameter of Fox R1 growth in control and test. Error bar represents the standard deviation.

**Figure 4.**
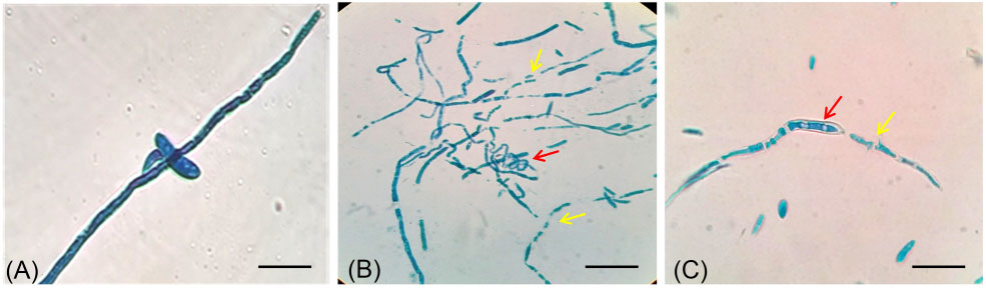
Microscopic images of Fox R1 grown in presence of Bar D5 volatiles. (A); control, (B) and (C), test. Structural deformities were observed as indicted by arrows. Yellow arrow indicates the fragmentation of hyphae whereas red arrow in (B) and (C) represents the coiling of hyphae and swelling of hyphae, respectively. Each bar represents 50 µm.

Further, Bar D5 was accessed for the production of antifungal volatiles by the seal plate method. The VOCs produced by Bar D5 not only inhibited the growth of Fox R1 but also caused degenerative changes in the morphology. The diameter of growth of Fox R1 in control was 8.4 ± 0.2 cm, and in test, the diameter was 6.49 ± 0.12 cm (Fig. 3E). A 22.7% (*P* = .00) reduction in zone due to inhibition of growth was observed after 7 days of incubation. In addition, a strong reduction in the purple pigment was observed. The Fox R1 in the presence of volatiles of Bar D5 appeared off-white compared to the control (Fig. 3A and B). The cottony texture of Fox R1 was also not observed in the presence of volatiles of Bar D5 compared to the control (Fig. 3C and D). Further, on microscopy, structural deformities were observed such as coiling of hyphae (indicated by the red arrow in Fig. 4B), fragmentation of hyphae (indicated by the yellow arrows in Fig. 4B and C), swelling of hyphae, and shrinkage of cytoplasm (indicated by the red arrow in Fig 4C) as compared to control (Fig. 4A). This indicated that Bar D5 has the potential for the production of VOCs that have an inhibitory effect on Fox R1.

### *In planta* antagonism of Bar D5 against Fox R1 in saffron corm in real time

*In planta* antagonism of Bar D5 against Bar D5 was studied using three approaches simultaneously: real time imaging, qPCR and culture-based method.

#### Real time imaging of saffron corms infected with EGFP-labeled *F. oxysporum* R1

**Figure 5.**
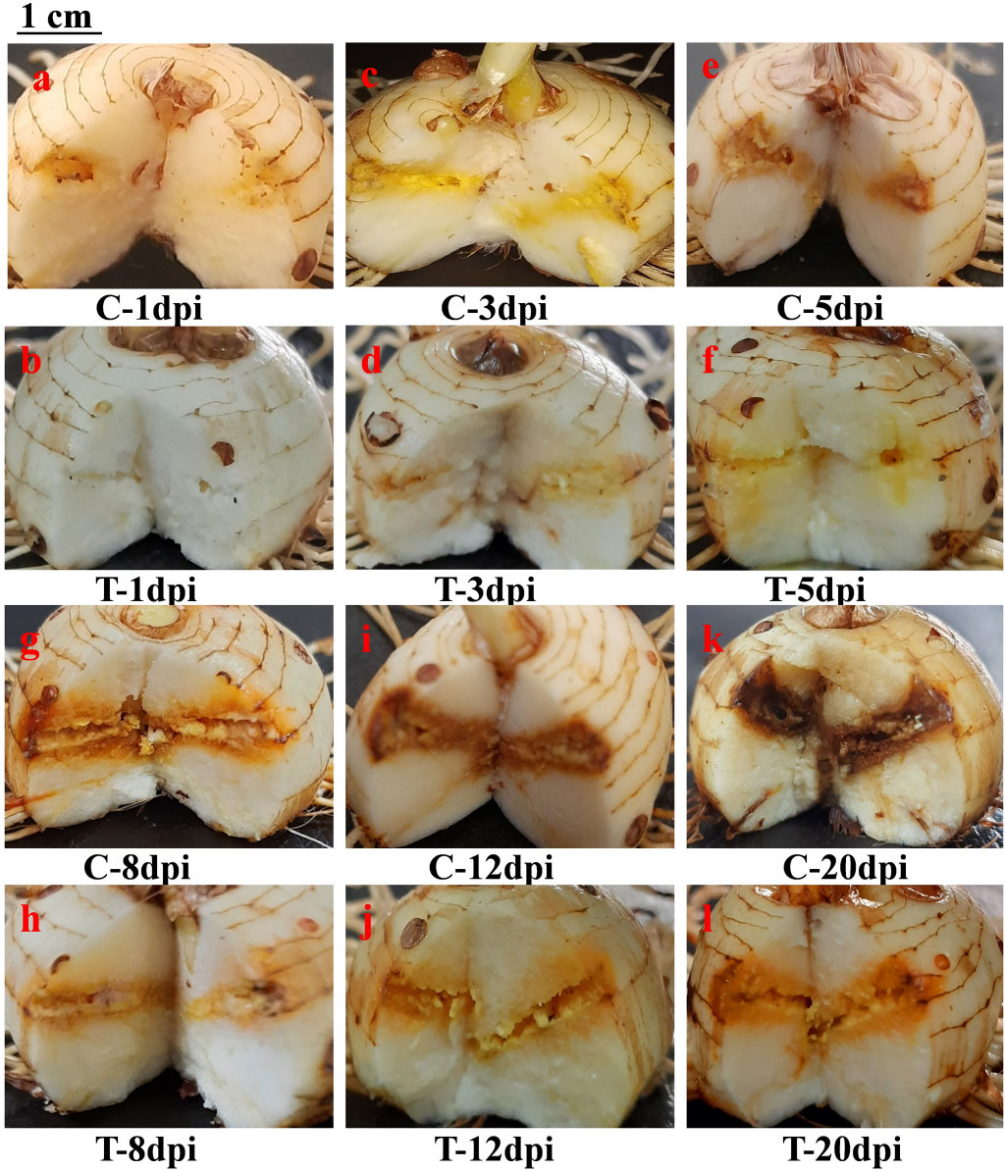
Representative pictures of saffron corm infected with Fox R1. C—control; mock-primed corms infected with Fox R1. *T*-test; Bar D5-primed corms infected with Fox R1. dpi—stands for days postinoculation.

The infected corms were dissected and observed for the visual symptoms caused by Fox R1 in control and test. The difference in the intensity of the symptoms was observed in the presence and absence of Bar D5 (Fig. 5). In control, the symptoms were more intense and ranging from yellowing to dark browning of the corms around the injury point. However, in presence of Bar D5, the symptoms of the corm rot were less intense (Fig. 5).

**Figure 6.**
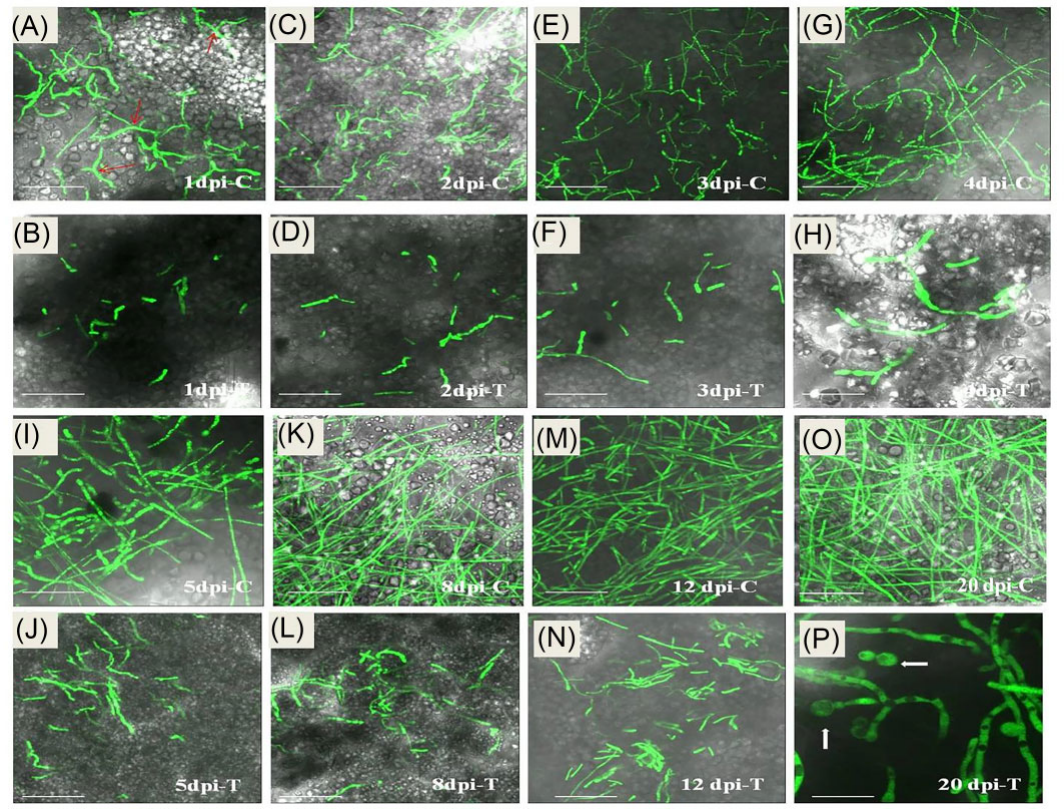
Confocal images of the corm tissue at different dpi under the confocal microscope at 60X. C—mock-primed corms inoculated with EGFP-tagged Fox R1, T—bar-primed corms inoculated with Fox R1 at different dpi. (A, C, E, G, I, K, M, and O) are the control images and (B, D, F, H, J, L, N, and P) are the test images. In image (p) arrow indicates the chlamydospores formed at 20 dpi in test. Each bar represents 10 µm scale.

Further, the thin sections of the infected corms tissue were visualized under the confocal microscope at 1, 2, 3, 4, 5, 8, 12, and 20 dpi. At 1 dpi, in mock-primed corms, Fox R1 hyphae were higher in number and extended compared to Bar D5 primed wherein very few and distorted hyphae were observed (Fig. 6A and B). At 2 and 3 dpi, in mock-primed corms the Fox R1 were growing optimally, however, in Bar D5-primed corms at 2 and 3 dpi comparatively fewer hyphae were observed. The hyphae were very short and swollen compared to the control at 3 dpi (Fig. 6C–F). A similar trend was observed at 4, 5, 8, and 12 dpi in control, where hyphae were elongated and in test samples less in number, shrunken and stressed (Fig. 6G–N). Interestingly, at 20 dpi in the test sample, the formation of chlamydospores occurred, as indicated by the arrows (Fig. 6P).

#### Quantification of Fox R1 load in saffron corm

**Figure 7.**
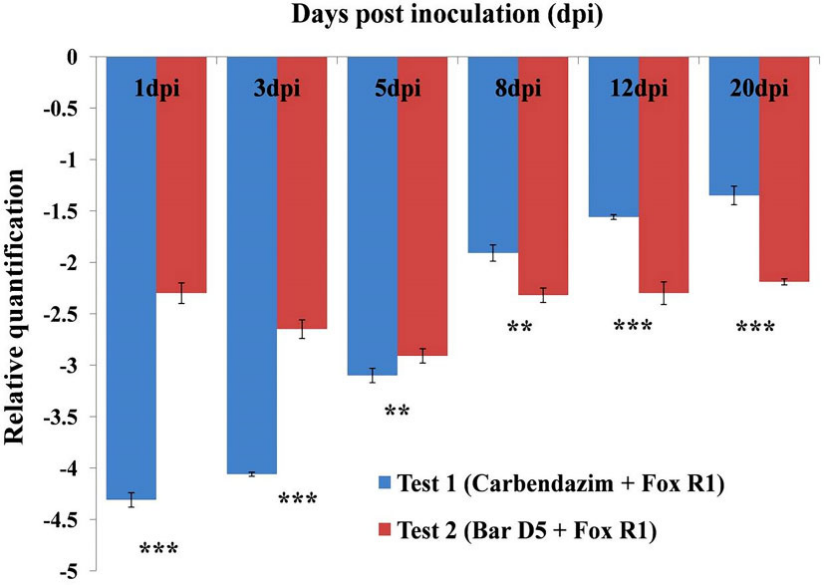
Relative quantification of Fox R1 in different treatments using Fox R1 specific primers. Results are expressed as mean ± standard deviation (*n* = 3). ***represents the significance level (*P* < .001) ** represents the significance level (*P* < .01)

Quantification of Fox R1 load in the absence and presence of Bar D5 was done in saffron corms by real time PCR. Since confocal microscopy images represent qualitative data; quantification of Fox R1 was done to get a complete picture. Fox R1 load was quantified in control, test 1 and test 2 using Fox R1 specific ITS primers. Test 1 was taken into consideration to draw a comparison between biocontrol Bar D5 and the chemical fungicide carbendazim to compare the effect of biocontrol with the commonly used fungicide. Up to 5 dpi the load of Fox R1 was less in test 1, i.e. carbendazim-treated corms as it was 4.31-, 4.06-, and 3.1-fold less compared to the control (Fig. 7). However, at 8, 12, and 20 dpi Fox R1 load was least in test 2 i.e. Bar D5-primed corms, the load was 2.38-, 2.3-, and 2.29-fold less compared to the control but in test 1 the load was 1.85-, 1.55-, and 1.5-fold less compared to control (Fig. 7). This indicated that Bar D5 has a longer-term effect compared to commercial fungicides.

#### Fox R1 load quantification in saffron corms under different treatments by CFU method

**Figure 8.**
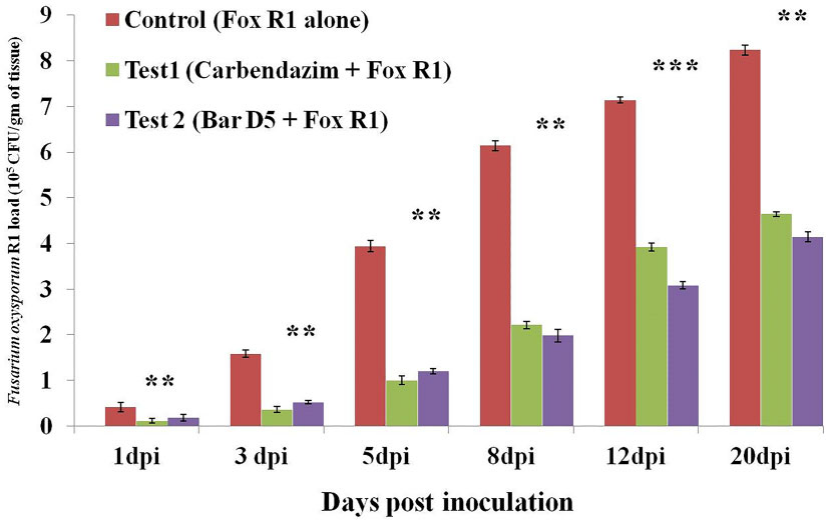
Absolute quantification of Fox R1 in different treatments using Fox R1 specific media by CFU method. Results are expressed as mean ± standard deviation (*n* = 3). *** represents the significance level (*P* < .001) ** represents the significance level (*P* < .01).

This method was done to complement and validate the results obtained from confocal microscopy and q-PCR. The results obtained were in sync with real time PCR data. In control, the load of Fox R1 kept on increasing up to 20 dpi. The maximum inhibition of Fox R1 was 74.6% and 70.0% at 5 dpi in test 1 and test 2, respectively, compared to the control (Fig. 8). In test 1, the load of Fox R1 was less compared to test 2 up to 5dpi. After 5 dpi, the load of Fox R1 increased in test 1 and was higher as compared to test 2. At 8, 12, and 20 dpi, the maximum inhibition was recorded in test 2 compared to pathogen control (Fig. 8). In the test 1, the inhibition percentage was 73% at 1 dpi but 43.6% at 20 dpi. However, in test 2, at 1 dpi the inhibition was 50.1% and 49.7% at 20 dpi. This indicated that the Bar D5 on average reduced the Fox R1 load by 50% in infected corms.

### Transcriptomic profiling of Fox R1 in Bar D5-primed corms

**Figure 9.**
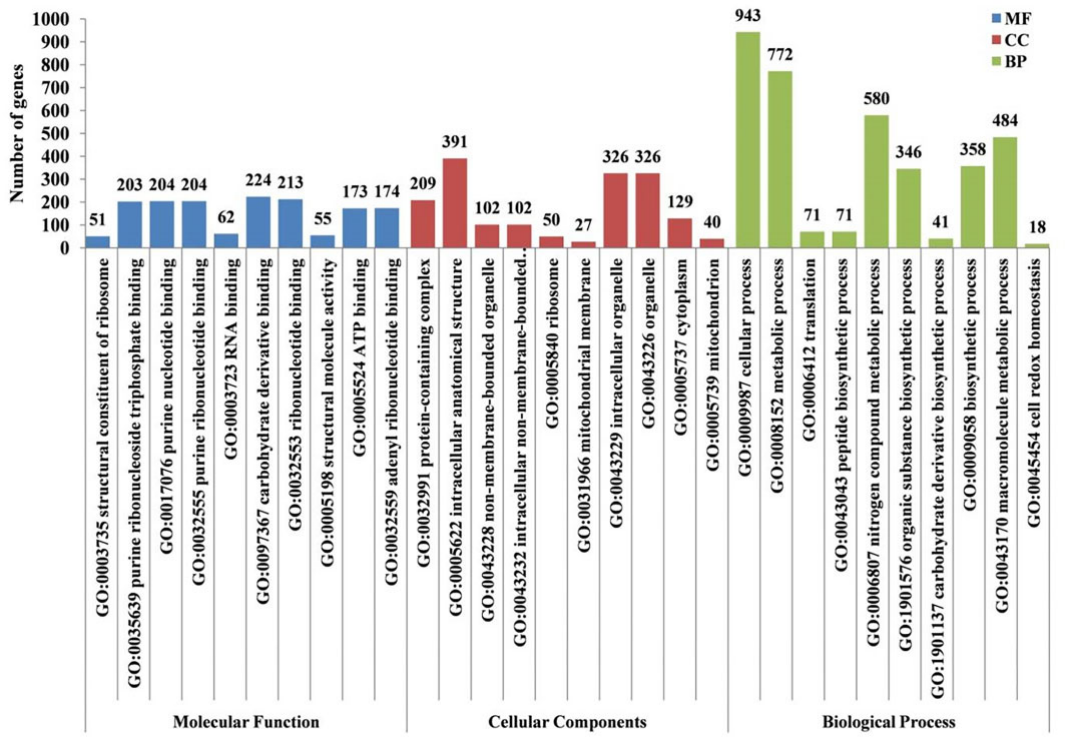
GO-enriched terms identified in Fox R1 under the influence of Bar D5 (C vs. T) by GO analysis. The GO terms are divided into three categories (molecular function, cellular components, and biological process) based on the function of the DEGs. The *y*-axis represents the number of DEGs and their positioning in *x*-axis is based on enrichment (adjp-value < .05).

To understand the molecular mechanism of action of Bar D5 against Fox R1, *in planta* transcriptomic analysis was performed to establish their interaction in a natural way. The expression of DEGs of Fox R1 was studied in control vs. test at 2 dpi. *In planta* interaction was conducted at 2 dpi as it represents the midpoint for interaction between initial time period i.e. 0 dpi to maximum defense response in saffron at 5 dpi. The raw reads obtained were processed for quality check and alignment with the reference genome of Fox, which resulted in 2.81% alignment in the control and 1.42% alignment of Fox R1 in the test sample. The number of raw reads mapped to the reference genome has been tabulated in Table 4. During the antagonism, 3689 significant genes based on (*P* < .05) of Fox R1 were expressed. A total of 1241 genes of Fox R1 were upregulated and 1282 genes were downregulated in presence of Bar D5. All the DEGs were subjected to GO functional annotation analysis. A total of 2553 GO terms were assigned of which 813 (31.8%) were related to molecular function, 1370 (53.6%) were related to biological process and 370 (14.4%) were related to the cellular component. The 96 significantly enriched GO terms (sorted by adjp-value < .05) were identified. The top 10 enriched GO terms in each category were plotted against the number of genes associated with each GO term (Fig. 9). The most enriched GO terms related to molecular function were structural constituent of ribosome (GO:0003735), purine ribonucleoside triphosphate binding (GO:0005198), in cellular component was protein-containing complex (GO:0032991) intracellular anatomical structure (GO:0005622), in biological process was cellular process (GO:0009987), and metabolic process (GO:0008152).

**Table 4.** The number of reads obtained in each sample and the % alignment obtained with *F. oxysporum* f.sp*. lycopersici* reference genome.

Sample ID	Description	Alignment rate	Average	Total reads (in millions)	Mapped reads of Fox R1	Unmapped reads of saffron corm
Control 1	Fox R1-inoculated corms	2.85	2.81	63 109 488	1800 727	61 308 761
Control 2		2.77		58 194 242	1613 203	56 581 039
Tet1 1	Bar D5-primed and Fox	1.24	1.425	85 792 446	1062 007	84 730 439
Test 2	R1-inoculated corms	1.61		75 464 060	1216 406	74 247 654

**Figure 10.**
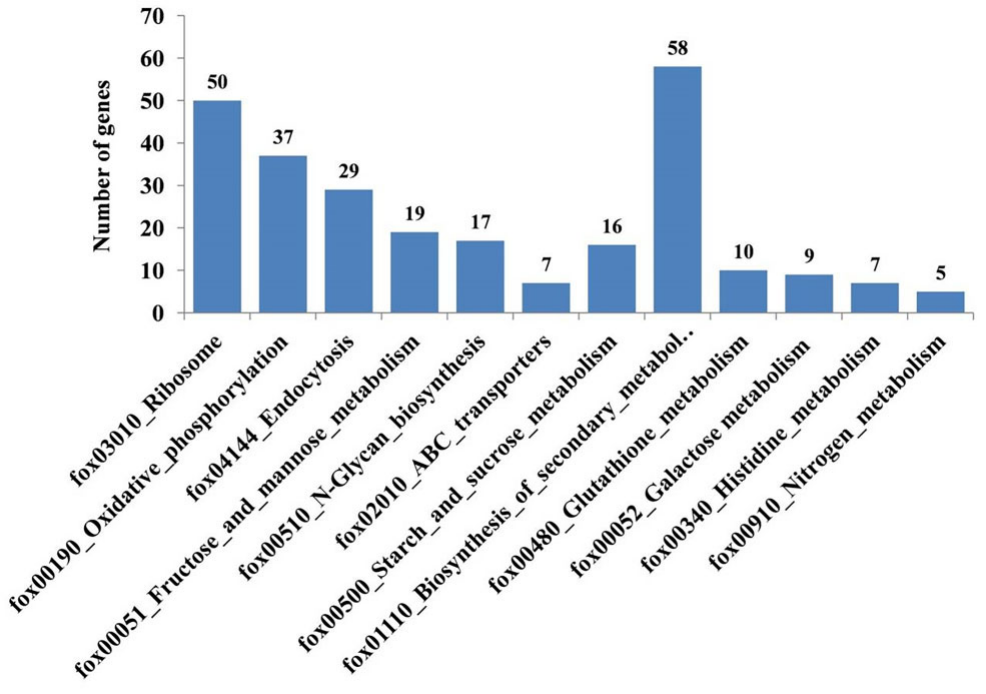
Enriched KEGG pathways identified in Fox R1 under the influence of Bar D5 (C vs. T). The *y*-axis represents the number of DEGs involved in particular pathway and their positioning on *x*-axis is on the basis of (adj *P* < .05).

Further, various metabolic pathways activated under the influence of Bar D5 were investigated using KEGG analysis. In total, 12 KEGG pathways were found to be enriched based on a significant *P-*value (*P* < .05). The top 10 enriched pathways were ribosome (fox03010), protein processing in endoplasmic reticulum (fox04141), oxidative phosphorylation (fox00190), fructose and mannose metabolism (fox00051), *N*-glycan biosynthesis (fox00510), biosynthesis of secondary metabolites (fox01110), starch and sucrose metabolism (fox00500), glutathione metabolism (fox00480), galactose metabolism (fox00052), and ABC transporter (fox02010) (Fig. 10). The pathway biosynthesis of secondary metabolites (fox01110) contained the maximum number of genes followed by the ribosome (fox03010) and protein processing in endoplasmic reticulum (fox04141).

### DEGs of Fox R1 essential for its growth, development, and pathogenicity affected by Bar D5

The genes of Fox R1 that are related to growth, morphology, and pathogenicity were selected for further analysis. A total of 60 such genes were identified by subjecting the total data to Pathogen–Host-Interaction database. Among 60 genes, 48 genes were found to be significant with *P* < .05. A total of 40 genes were found to be differentially expressed with log2 fold change ≥ 0.5 and 23 genes were found to be differentially expressed with log2 fold change ≥ 1 in control vs. test. All the 60 genes identified have been enlisted in the Table 5.

**Table 5.** List of *F. oxysporum* R1 genes effected by biocontrol agent *Bacillus* sp. strain D5.

Gene ID	Category	Gene description	Log2 fold (C vs. T)	*P*-value
FOXG_04179	Cell wall synthesis	Chitin synthase D	−0.50	.032
FOXG_00113		Chitin synthase 4	−1.94	.03
FOXG_04162		Chitin synthase V	−0.67	.00057
FOXG_03721		1,3-beta-glucan synthase	−0.5	.037
FOXG_10530	Plasma membrane	Ergosterol biosynthesis protein	−0.3	.027
FOXG_04155		Phospholipase A2	−1.2	.03
FOXG_03915	ROS detoxification	Catalase	−0.8	.006
FOXG_05962		Glutathione S transferase	−7.4	1.23E-05
FOXG_08382		Glutathione S transferase	0.3	.008
FOXG_08657		Epoxide hydrolase	−7.1	4.08E-05
FOXG_00069		Thioredoxin	−0.37	.002
FOXG_00831		Thioredoxin reductase	−0.47	.0001
FOXG_01626	Nitrogen metabolism	Glutamate dehydrogenase	−1.16	.004
FOXG_05182		Glutamine synthetase gene	1.5	.07*
FOXG_10292	Carbon metabolism	Alcohol dehydrogenase	−5.25	.02
FOXG_12868	Transporters	ABC transporter	−7.8	1.62E-06
FOXG_13653		ABC transporter CDR4	−1.89	.3*
FOXG_01943		ABC multidrug transporter	0.7	.007
FOXG_13215		MFS transporter	−1.99	.002
FOXG_15621			−0.92	.09*
FOXG_15370			−1.80	.002
FOXG_03950		MFS toxin efflux pump	−7.6	4.12E-06
FOXG_15681		MFS peptide transporter	−6.5	.0003
FOXG_19077	Plant cell wall degrading enzymes	Pectin lyase	−0.82	1.97E-06
FOXG_12330		Pectinmethylestrase	0.82	2.66E-09
FOXG_13051		Endopolygalacturonase	−0.6	.018
FOXG_08862		Exopolygalacturonase	1.08	0.0002
FOXG_15742		Endo-beta-1,4-xylanase	−7.8	3.89E-05
FOXG_02322	Mycotoxin production	Fusaric acid	0.76	0.04
FOXG_03748	*xnlr*	Zn(II)2Cys6 TF	−11.4	3.36E-15
FOXG_06378	*fow2*	Zn(II)2Cys6 TF	0.17	.13*
FOXG_04196	*ctf1*	Zn(II)2Cys6 TF	0.07	.86*
FOXG_05408	*ebr1*	Zn(II)2Cys6 TF	−2.2	.02
FOXG_02103	*ste12*	C2H2 TF	−0.18	.8*
FOXG_11503	*con7-1*	C2H2 TF	0.5	.003
FOXG_00370	*zafa*	C2H2 TF	0.4	.2*
FOXG_02277	*meab*	bZip TF	0.7	.04
FOXG_03165	*fnr1*	GATA TF	0.66	.045
FOXG_01993	*snt2*	PHD TF	−3.7	.01
FOXG_10510	*sge1*	Gti1/Pac2 family	−1.27	.0007
FOXG_10430	*ren1*	HSF TF	1.83	.005
FOXG_09390	*ftf2*	Fusarium transcription factor-2	−2.80	2.04E-05
FOXG_11273	*vea*	Velvet family	3.4	.01
FOXG_00016	*velb*		−0.21	.4*
FOXG_08140	*fmk1*	MAP kinase	0.26	.08
FOXG_06318	*hog1*		0.11	.3*
FOXG_03107	*pbs2*		−4.9	.001
FOXG_09359	*fga1*	Guanine nucleotide-binding protein alpha subunit	2.05	.1*
FOXG_11532	*fgb1*	Guanine nucleotide-binding protein beta subunit	−0.69	.0001
FOXG_09254	*msb2*	Mucin rich membrane protein	−0.29	.8*
FOXG_06120	*sho1*	Tetraspan membrane protein	−0.58	.002
FOXG_00386	*rho1*	Monomeric G-protein	−0.37	.02
FOXG_00058	*frp1*	F-box protein required for pathogenicity	0.3	.4*
FOXG_01957	*arg1*	Arginosuccinate lyase	0.38	.008
FOXG_01489	*cnb1*	*Calcineurin* subunit B	1.13	.02

* Nonsignificant genes in C vs. T.

TF—transcription factor.

MAP kinase—mitogen-activated protein kinase.

ROS—reactive oxygen species.

**Figure 11.**
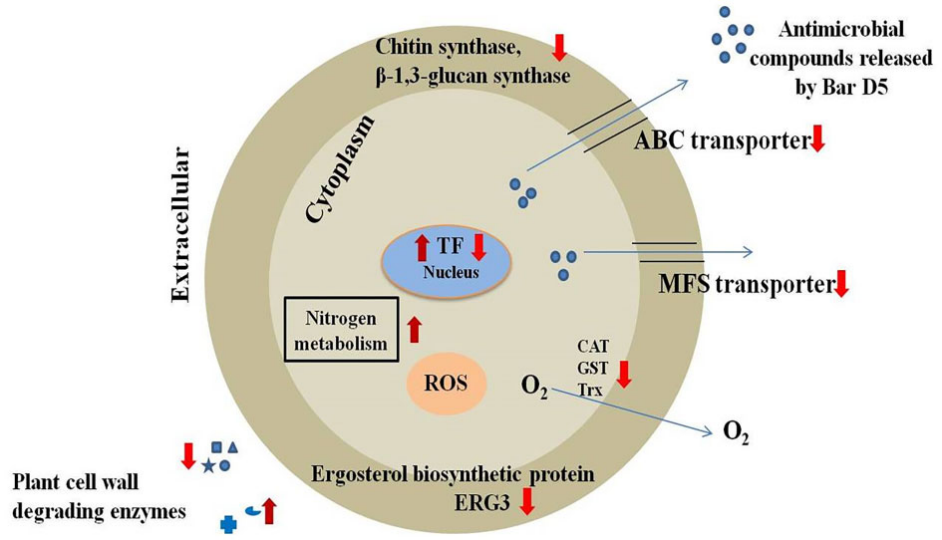
Pictorial representation of gene/pathways of Fox R1 affected by Bar D5. ↓ Indicated downregulated of genes and indicated ↑ upregulation of genes.

A total of 3 DEGs encoding different chitin synthase enzymes of Fox R1 were found to be downregulated by Bar D5, which includes chitin synthase D (FOXG_04179), chitin synthase 4 (FOXG_00113), and chitin synthase V (FOXG_04162). Another important gene encoding 1,3-beta-glucan synthase (FOXG_03721) responsible for cell wall construction was significantly downregulated in presence of Bar D5 including erg3 (FOXG_10530), and phospholipase A2 (FOXG_04155) (Table 5) (Fig. 11). The DEGs related to reactive oxygen species (ROS) detoxification were identified such as catalase (FOXG_03915), glutathione S-transferase (FOXG_05962), thioredoxin (FOXG_00069), epoxide hydrolyse (FOXG_08657), and peroxiredoxin. In the present study, gene encoding peroxiredoxin of Fox R1 was upregulated in presence of Bar D5; however, genes encoding other enzymes were downregulated (Table 5).

The gene of Fox R1 encoding alcohol dehydrogenase (FOXG_10292) was downregulated that plays important role in the carbon and energy metabolism of fungi. In contrast, the enzymes such as glutamine synthetase (FOXG_05182), transcription factors (TFs) Fnr1 (FOXG_03165) were upregulated in presence of Bar D5 (Table 5). Further, two DEGs related to ATP-binding cassette (ABC) transporter and five genes related to major facilitator superfamily (MFS) transporter were downregulated. The gene encoding MFS toxin efflux pump (FOXG_03950) and MFS peptide transporter (FOXG_15681) was also downregulated in presence of Bar D5. Bar D5 significantly downregulated the expression of genes encoding for plant cell wall degrading enzymes (PCWDEs) such as pectin lyase (FOXG_19077), xylanase (FOXG_15742), and endo polygalacturonase (FOXG_13051). However, the expression of two genes encoding exo polygalacturonase (FOXG_08862) and pectin methyesterase (FOXG_12330) were significantly upregulated. The various gene encoding different TFs in Fox R1 was downregulated in presence of Bar D5. The expression of genes for TF Ebr1 (FOXG_05408), Xnlr (FOXG_03748), Sge1 (FOXG_10510), Snt2 (FOXG_01993), and FTF2 (FOXG_09390) were significantly downregulated in presence of Bar D5. In addition, the DEG encoding MAP kinases Pbs2 (FOXG_03107) was significantly downregulated but the expression of two other genes encoding MAP kinases [Hog1 (FOXG_06318) and Fmk1 (FOXG_08140)] was found nonsignificant. Another important gene *cnb1* (FOXG_01489) that encodes regulatory subunit B of calcineurin, a heterodimeric calcium/calmodulin dependent protein phosphatase involved in the regulation of chlamydospores formation was upregulated (Table 5).

### Validation of transcriptomics data by q-PCR

**Figure 12.**
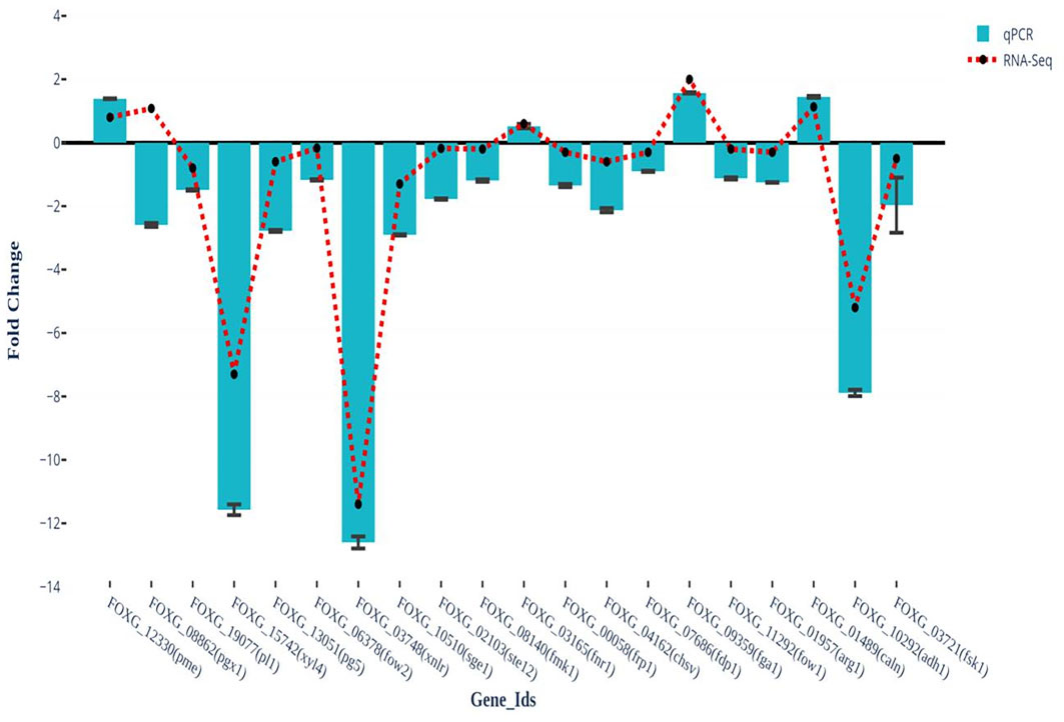
Validation of the expression patterns of genes of Fox R1 selected from transcriptome by RT-qPCR. Gene expression values in different comparisons were obtained by normalizing the values. Fox R1 actin gene was used as RG. Values are expressed as mean ± sd (*n* = 3).

In total, 20 genes were selected from the transcriptome data for validation using q-PCR. A total of six genes encoding for TF, five genes encoding for PCWDEs, two genes encoding for G-proteins and cell wall synthesizing enzymes each, one gene encoding for each arginosuccinate lyase, mitochondrial carrier proteins, calcineurin B subunit, and alcohol dehydrogenase enzyme (Fig. 12). The actin gene was used as the RG for the normalization of data. The effect of Bar D5 on the genes of Fox R1 was evaluated using **∆∆**Ct method. The results of q-PCR were consistent with the RNA seq data except or one gene, i.e. *pgx1* gene that has shown upregualtion in RNA seq data but downregulation in q-PCR results. The similar expression pattern of the remaining 19 genes supports the results of RNA seq (Fig. 12).

## Discussion

Corm rot of saffron caused by the pathogen *F. oxysporum* is one of the most devastating diseases that affect its production worldwide (Mansotra et al. 2023). The disease spreads from the sowing of injured, wounded, or infected corms in soil infested with *F. oxysporum* as its chlamydospores can persist in soil for many years (Gupta et al. 2021, Bhagat et al. 2022, Mansotra et al. 2023). Although chemical management is employed for disease management, in recent years attention has shifted to the use of an ecofriendly approach i.e. the use of biocontrol agents (Mawar et al. 2021). Biocontrol agents employ multiple strategies for disease control, such as competition for food and niches, the secretion of inhibitory compounds, and the induction of systemic resistance in the host plant (Boro et al. 2022).

Although Bar D5 exhibited mild antifungal activity in dual culture, it has been reported to decrease the disease incidence in pot trials by 71% and by 57% in field trials (Magotra et al. 2021). In the present study, Bar D5 was demonstrated to secrete volatiles in the seal plate assay, as indicated by the growth inhibition and change in morphology of Fox R1. Previously, two genes for volatile compounds (VOCs) such as 2, 3 butanediol and acetoin were identified from the draft genome of Bar D5 (Magotra et al. 2021). VOCs produced by *Bacillus* species such as *Bacillus* sp. strain B44 and *Bacillus cereus* MH778713 have been reported to inhibit the growth of disease causing *F. oxysporum*, hence acting as antifungal compounds (Gotor-Vila et al. 2017, Jangir et al. 2018, Ramirez et al. 2022).

Additionally, the cell-free extract of Bar D5 caused structural deformities in Fox R1 (Fig. 2). Structural deformities in Fox R1 have also been reported by cell-free extracts of *B. amyloliquefaciens* W2 and *Burkholderia gladioli* E39CS3 (Gupta and Vakhlu 2015, Ahmad et al. 2022). The cell-free extracts of various species belonging to the genera *Bacillus, Streptomyces*, and *Tricoderma* have been reported to cause structural abnormalities in various phytopathogens (Kubicek et al. 2001, Trejo-Raya et al. 2021). The effect of Bar D5 was evaluated against Fox R1 in Bar D5-primed saffron corms, and chlamydospore formation of Fox R1 was observed at 20 dpi, indicating the fungistatic mode of action of Bar D5 (Fig. 6P). Chlamydospores are asexual resting cells that are formed in response to stress. Warma and Aitken (2018) have reported the formation of chlamydospores of *F. oxysporum* in the decaying leaf sheath of two different varieties of banana, Lady Finger and Cavendish at 70 dpi and 80 dpi, respectively.

Further, to check the effect of the D5 on the growth of Fox R1 *in planta*, the load of Fox R1 was determined in the absence and presence of Bar D5 in saffron corm, and it was observed that up to 5 dpi carbendazim was better, but after 5 dpi, Bar D5 efficiently reduced the load of Fox R1 in infected samples (Fig. 7). The results of Fox R1 load in the presence and absence of biocontrol were further complemented by the CFU method and a similar trend of inhibition of Fox R1 was observed (Fig. 8). *Bacillus subtilis* SQR9, *Trichoderma asperellum*, and *B. amyloliquefaciens* sub sp. *plantarum* XH-9 have also been demonstrated to reduce the load of *F. oxysporum in planta*, (Cao et al. 2011, Saravanakumar et al. 2016, Wang et al. 2018). In the present study, carbendazim was effective in controlling Fox R1 initially, but Bar D5 was found to be a better management strategy in the long run (Fig. 7 and 8). Similar to the present study, Gupta et al. (2020) have demonstrated the effects of different biocontrol agents and carbendazim against *F. oxysporum* causing corm rot in saffron fields. After 250 days of corm sowing, only 4% inhibition was reported in carbendazim treated samples, but 80% with the biocontrol agent *T. asperellum*. This clearly indicated that biocontrol agents are better in the long run compared to commercial fungicides.

### Interaction of Bar D5 and Fox R1 at transcriptome level

To unravel the interaction of Bar D5 and Fox R1 at the molecular level, the transcriptomes of *F. oxysporum* R1 in the absence and presence of Bar D5 in the saffron corm were compared. The results of transcriptomics confirmed that Bar D5 affects the growth, morphology, and pathogenicity profiling of Fox R1 as genes of Fox R1, related to these were downregulated in presence of Bar D5 (Fig. 11).

It was observed that a number of genes related to cell wall synthesis, cell membrane, carbon metabolism, and plant cell wall degradation were downregulated in Fox R1 in the presence of Bar D5. In the comparative transcriptomics analysis, three DEGs encoding different chitin synthase (CHSs) enzymes of Fox R1 were downregulated (Table 5). CHSs are the key enzymes involved in chitin synthesis and can also trigger an innate immune response in host plant (Li et al. 2016). Another important gene encoding 1, 3-beta-glucan synthase, was significantly downregulated in the presence of Bar D5. 1, 3-beta-glucan synthase is a glucosyltransferase enzyme that is involved in the synthesis of beta-glucan, a major component of fungal cell walls (Ruiz-Herrera and Ortiz-Castellanos 2019). The fungal plasma membrane, present next to the cell wall, is the place where multiple cellular processes of importance occur (Athanasopoulos et al. 2019). Ergosterol is one of the major components of the cell membrane of fungus, plays an important role in the permeability and fluidity of the membrane, and was downregulated in the present study (Rodrigues 2018, Mohid et al. 2022). The *erg3* gene has been reported to be involved in nutritional differentiation and virulence regulation in *Fusarium graminerum* (Yun et al. 2014). Similar to the present studies, researchers have also reported the downregulation of these genes in many fungal pathogens in the presence of biocontrol agent (Han et al. 2021). In contrast to the present study, in response to the biocontrol agent *B. amyloliquefaciens* the genes related to cell wall synthesis and cell membrane was significantly upregulated in *Sclerotinia sclerotiorum*, suggesting a robust defense response of this pathogen (Yang et al. 2020).

Another downregulated gene in the present study is phospholipase A2 (Table 5), an important enzyme that is involved in the hydrolysis of phospholipids into various signaling molecules such as phosphatidic acid, free fatty acids, diacylglycerol, and so on. These signaling molecules regulate diverse processes in cells such as growth and development, homeostasis, signal transduction, and so on in fungi (Zhu et al. 2016, Barman et al. 2018). The subfamily A2 of phospholipase has been reported to play an important role in signal transduction, homeostasis, and virulence (Roy et al. 2021).

Moreover, catalase- and glutathione-encoding genes were significantly downregulated in the presence of Bar D5. The catalase enzyme is a type of antioxidant enzyme that regulates the production of free radicals. It breaks down the hyderogen peroxide into oxygen and water molecules (Valenzuela-Cota et al. 2019). The glutathione-encoding gene encodes antioxidant enzymes that catalyze the conjugation of glutathione to various nonpolar molecules (Dhokane et al. 2016). Two genes for thioredoxin and one for thioredoxin reductase were also downregulated. Thioredoxins are ubiquitous proteins that reduce oxidized cysteine residues by cleaving disulfide bonds, protecting proteins from oxidative aggregation and inactivation. Thioredoxin reductase regulates intracellular oxidative potential by reducing thioredoxins (Zhang et al. 2020). However, in the present study, one gene for peroxiredoxin was upregulated. Peroxiredoxin also plays an important role by regulating the peroxide level in the cell. In the presence of Bar D5, overall the antioxidative potential of Fox R1 is hampered, as documented by the downregulation of various genes involved in the detoxification of ROS (Table 5) (Fig. 11).

Nitrogen is one of the essential elements required for the growth and development of fungi and enables them to survive nutrition depletion and different environmental niches (Tudzynski 2014). The expression of TFs Fnr1 (Fusarium nitrogen regulator-1) that regulate nitrogen catabolism and pathogenicity in *F. oxysporum* was upregulated in the presence of Bar D5. In addition, two genes encoding two main enzymes involved in the nitrogen metabolism pathway, i.e. glutamate dehydrogenase and glutamine synthetase, were identified. However, gene encoding glutamine synthetase was upregulated and gene encoding glutamate dehydrogenase was downregulated. In addition to nitrogen, another key factor for growth and development is carbon. The gene encoding alcohol dehydrogenase was highly down regulated (Table 5). Alcohol dehydrogenase plays a central role in carbon and energy metabolism and is necessary for fungal infection in plants. The downregulation of this gene implies that the carbon metabolism of Fox R1 has been negatively affected by Bar D5. Expression of the *F. oxysporum adh1* gene is reported to occur mainly during the initial periods of colonization of the plant, as during the invasion of roots, this gene helps *F. oxysporum* survive under hypoxia conditions (Escobosa et al. 2011).

The toxic compounds produced by the host or other microorganisms in the vicinity of the fungi hamper the growth of the fungi. These toxins, when accumulated in the cell, inhibit the growth and morphology of fungi. The toxins are removed from the cell with the help of transporters that efflux the antifungal compounds (Perlin et al. 2014). The two transporter families involved in efflux are the ABC superfamily and the MFS. ABC transporters function in the efflux of toxic compounds and fungicides and are also the center for nutrient transport (Fravel et al. 2003, Kumari et al. 2021). MFS, the main promoter super family of membrane transporters, is a transporter that is more selective than ABC transporters (Colemam and Mylonakis 2009). Two genes of Fox R1 related to the ABC transporter and five genes related to the MFS transporter were downregulated (Table 5). The genes encoding the MFS toxin efflux pump and MFS peptide transporter were totally repressed in the presence of Bar D5. The downregulation of DEGs related to ABC and MFS transporters has also been reported in *F. oxysporum* by *B. subtilis* HSY21 (Han et al. 2021). However, upregulation of DEGs related to the ABC transporter in *S. sclerotiorum* and *Fusarium pseudograminearum* in response to *Bacillus* revealed the robust response of the pathogen (Yang et al. 2020, Zhang et al. 2022).

### Effect of Bar D5 on the pathogenicity profiling of Fox R1

Bar D5 has significantly downregulated the expression of genes encoding PCWDEs, such as lipase, alpha-amylase, pectin lyase, xylanase, and endopolygalacturonase (Table 5). However, the expression of two genes encoding exopolygalacturonase and pectinmethyesterase was significantly upregulated. All these enzymes play an important role in virulence, as there is a correlation between PCWDEs and the pathogenicity of fungi. Similar to the present study, Zhang et al. (2022) have also reported the downregulation of genes of *F. pseudograminearum* encoding PCWDEs except for pectinmethyesterase, which was upregulated in presence of the biocontrol agent *Bacillus velezensis* YB-185. The downregulation of various CWDEs of *F. oxysporum* (soyabean root rot agent) has been reported by biocontrol agent *B. subtilis* HSY21 (Han et al. 2021).

Further, the various gene encoding TFs in Fox R1 were found to be downregulated in the presence of Bar D5. The genes encoding TFs Ebr1, Xnlr, Sge1, Snt2, and FTF2 were significantly downregulated in the presence of Bar D5. All these TFs have an important role in the virulence and pathogenicity of different f.sp. of *F. oxysporum*. The TF Xnlr regulates the expression of the gene encoding PCWDE xylanase (Zuriegat et al. 2021). The TF Sge1 regulates the invasive growth, virulence, and activities of extracellular amylase and cellulase in *F. oxysporum* f.sp. *lycopersici* (Rispail and Di Pietro 2010) and Snt2 regulates the growth parameters such as hyphae growth, septation, and conidiation in *F. oxysporum* f.sp. *melonis* (Denisov et al. 2011). The downregulation of these genes implies that the infection efficiency of Fox R1 is hampered. The gene encoding MAP kinases (Pbs2) was significantly downregulated; however, the expression of genes encoding MAP kinases (Hog1 and Fmk1) was found to be nonsignificant. The MAP kinase Fmk1 and Hog 1 play an important role in the pathogenicity of *F. oxysporum*, as Fmk1 regulates the growth, colonization, and expression of genes involved in cell wall synthesis, while Hog 1 is involved in oxidative stress and osmolarity response of *F. oxysporum* (Pareek and Rajam 2017).

Another important gene *cnb1* was found to be upregulated and encodes regulatory subunit B of calcineurin, a heterodimeric calcium/calmodulin-dependent protein phosphatase involved in the regulation of chlamydospores formation and the virulence of *F. oxysporum* f.sp. *lycopersici* (Hou et al. 2020). The upregulation of *cnb1* gene can be correlated with the formation of chlamydospores both in *in vitro* and *in planta* experiments.

The comparison of the DEGs in *F. oxysporum* R1 in the presence and absence of biocontrol clearly indicates downregulation of the genes of pathogens important for growth, morphology, and pathogenicity and upregulation of genes involved in nitrogen metabolism. Overall, the negative effect of Bar D5 on Fox R1 is confirmed by the results of comparative transcriptomics, q-PCR and CFU/g of infected corm quantification.

## Conclusion

Though biocontrol agents are used to control plant diseases for sustainable cultivation, a detailed study of the mechanism of their action on pathogenic agents is lacking altogether in some cases, such as the tripartite interaction between saffron-Fox R1 and Bar D5. In the present study, the effect of Bar D5 on the growth of the pathogenic Fox R1 has been clearly demonstrated *in vitro* as well *in planta*. Further, the genes up and downregulated have also been identified. This work can be further complimented by the studies, wherein selected fungal genes from the DEGs will be mutated, and the effect of this mutation will be checked on its ability to cause disease.

## Supplementary Material

xtad025_Supplemental_File

## Data Availability

The transcriptome sequencing data has been submitted to NCBI under the accession numbers PRJNA938757 and PRJNA938758.
