# Supplementary material for: Effects of biocontrol Bacillus sp. strain D5 on the pathogenic Fusarium oxysporum R1 at the microscopic and molecular level in Crocus sativus L. (saffron) corm
Source: FEMS Microbes. 2024 Jan 3;5:xtad025. doi: 10.1093/femsmc/xtad025 (PMC10799715; doi:10.1093/femsmc/xtad025)
Supplement: xtad025_Supplemental_File [file xtad025_supplemental_file.docx]

**Reviews of manuscript FEMSMC-2023-006**

**Reviewer 1: Iqbal Ahmad, Aligarh Muslim University**

**Recommendation: Accept**

The authors have provided new information on the effect of biocontrol agent Bacillus sp

on fungal pathogens growth, activity and up and down regulation of various pathways genes indicating global impact on gene expression However secondary metabolism pathways are significantly affected.

This manuscript have provided an improved basis of understanding on the mode of action of biocontrol agent. It may lead to further mutation based analysis for further in depth and focused study on the mechanism of action. Overall it is well written. Discussion may be shortened.

**Reviewer 2**

**Recommendation: Major Revision**

In this work, Bhagat and Vakhlu characterize the biocontrol activity of Bacillus sp. strain D5 (Bar D5) against the well-known pathogen Fusarium oxysporum R1 (Fox R1) using different techniques in vitro and in vivo in saffron plants (Crocus sativus). The authors claim that this pathogen is a major problem for this crop and the disease caused by the pathogen, the corm rot, one of the main reasons to explain the progressive decline of its cultivation in India. Management of the disease is normally done with antifungal chemicals, with the subsequent negative effect in the environment. The authors propose that Bar D5 can be used in the future as a biocontrol agent to control the corm rot of saffron in replacement of antifungals.

Overall, this work describes extensively the inhibition of Fox R1 by Bar D5 and its protective effect over the plant, providing some novel insights with very simple and easy to understand experiments. The topic of the research proposed by the authors is interesting and their conclusions seem robust enough to merit publication. However, I have some concerns that, in the opinion of this reviewer, need to be addressed before publication, especially in some of the methods and in the way the data is presented.

Major comments:

-In the methods section, in general, the methods need to be properly described. A reference is not enough to explain a technique. The paper would benefit from a more complete description, especially, in the preparation of the bioformulation of the strain Bar D5. This should be explained in detail or at least, should be briefly described along with a reference of the exhaustive preparation technique. Also, In the q-PCR of used to quantify the pathogen load during infection, the article would benefit from a more detailed description regarding how the relative quantification was calculated. In addition, the authors chose as a reference gene a reference gene from the plant and not from the pathogen (the crocus 18S gene) is there any explanation? Seems more logical to use a gene from the pathogen as a reference, since the pathogen’s DNA is the one being quantified.

-Fig.1 shows the inhibition of Fox R1 by Bar D5. The data presented is qualitative, and consists only of images of the plates with the pathogen in the presence or absence of Bar D5. The authors claim that “Bar D5 inhibits the growth of Fox R1 by 41%” (page 9 line 18). This needs to be supported by quantitative data and its corresponding statistical analysis. Quantification of the diameter of growth of the pathogen can be done in image J using the pictures of the plates and the resulting values can be used to calculate and plot the % of inhibition with its corresponding statistical analysis.

-Authors claim that in their seal plate method to evaluate the production of volatile compounds by the bacterial colony, they observed a growth reduction of 25%. How is this percentage calculated? Based on what measurements? Quantitative data needs to be provided.

-Fig. 2 and 3: The microscopy images presented have very poor quality and cannot be published in their current state. In addition, some of the images (Fig. 2 A and D, Fig. 3 F) look like they have been manually enlarged. Also, the images do not have a scale bar. New and better images are required if these data and its discussion are going to be kept in the final text of the article. In addition, please, provide the original images.

-Additionally, there is some confusion in the figure legends of Fig. 2 and 3, which need to be more descriptive and carefully revised. Legend in Fig. 2 mentions “complete loss of pigmentation”, which is something that cannot be observed in Fig. 2. There are also reference in Fig.2 to e f g or h which are not part of Fig. 2. The legend of Fig. 3 a refers to the length of hyphae, which are not part of that figure. These are just some examples.

-The bright field and fluorescence images in Fig. 4 should be presented separately along with the overlay.

-The data from the quantification of the Fox R1 load by CFU counts in the infected plants treated or untreated with Bar D5 is summarized on a table. A plot would be a much more informative way of presenting these data and it will show the statistical validation. This plot could go in the same figure as the one in Fig. 5.

-Concerning the characterization of the antagonism of Bar D5 against Fox R1 in planta, it would be very helpful, if feasible, to have also pictures of the infected corms treated or non-treated with Bar D5 to see differences in disease progression and/or severity. This data could also be accompanied by scanning electron microscopy images showing a detail of the fungal growth in treated plants compared to the untreated control.

-Concerning the transcriptomic analysis of Fox R1 during infection in the presence of Bar D5, why did the authors choose 2 dpi as their condition? This needs to be better justified. Especially, considering that maximum inhibition is achieved at 5 dpi, according to the authors (page 10, line 52), which would also be an interesting time-point to study. Furthermore, what is the fold change threshold value used by the authors to consider a gene as differentially expressed?

-In section 3.4, the authors comment on some of the differentially expressed genes that they found in the presence of Bar D5 during the infection. The reader would benefit from a small introduction in this section to make the story a bit more cohesive. Why are we talking about these genes specifically our of 3689 differentially expressed genes, according to the authors? Why is the alteration of their gene expression in the presence of Bar D5 important? Part of this is mentioned in the discussion section, but it is this reviewer opinion that the discussion section is too long, and the flow of the article would benefit from mentioning some of these points during the results section. Some parts of the discussion are also too exhaustive (for instance, page 15 lines 25-41). I believe that this section should be better summarized (or even shortened), in general.

Minor comments:

-In the methods section, an accession number is provided for Bar D5 (KT228251) and the authors mention in page 4 line 7 a “draft genome sequence”. However, the accession number leads to a genbank page containing only the sequence of the 16S rRNA (https://www.ncbi.nlm.nih.gov/nuccore/KT228251).

-In the discussion section, the authors mention that “The results of q-PCR revealed that the Bar D5 was better compared to carbendazim in controlling the growth of Fusarium oxysporum R1” (page 16 lines 16-19), but this is inaccurate, as carbendazim is more efficient at earlier time-points post infection.

-Authors mention several times throughout the text the expressions “was more” or “was less” (for example in page 10 line 56). I believe a better wording would be “was higher” or “was lower” (increased or decreased are also possible options in some parts of the text).

**Author's Response to Decision Letter for (FEMSMC-2023-006)**

I would like to thank you on behalf of all the authors for considering our work in FEMS Microbes. The manuscript entitled “Mechanism of action of biocontrol Bacillus sp. strain D5 on the pathogenic Fusarium oxysporum R1 at microscopic and molecular level in Crocus sativus L. (saffron) corm” has been revised as per the suggestions of the reviewers.

The text revised in the manuscript is highlighted in red.

Kindly find the point wise response to the comments /suggestions raised by the reviewers.

Associate editor:

Comment: Both reviewers find this work valuable and of interest. However, Reviewer two raises several important concerns regarding the presentation of data, especially with regards to quantification and statistical analysis. The Reviewer also makes several helpful suggestions for improving the writing of the manuscript, presentation of additional details in methods, and so on. Thus, in its current form this manuscript is not suitable for publication. I encourage the authors to submit a revised version if they are able to satisfactorily address all of Reviewer 2’s comments and concerns.

Response: Thank you for considering our work. The manuscript has been revised as per the suggestions of the reviewers.

Reviewer #1:

Comment: The authors have provided new information on the effect of biocontrol agent Bacillus sp on fungal pathogens growth, activity and up and down regulation of various pathways genes indicating global impact on gene expression however secondary metabolism pathways are significantly affected. This manuscript has provided an improved basis of understanding on the mode of action of biocontrol agent. It may lead to further mutation based analysis for further in depth and focused study on the mechanism of action. Overall it is well written. Discussion may be shortened.

Response: Thank you for the critical evaluation and considering our work. As suggested the discussion part of the manuscript has been shortened. The suggestion regarding the mutation based studies will definitely be taken into consideration for future studies.

Reviewer 2:

In this work, Bhagat and Vakhlu characterize the biocontrol activity of Bacillus sp. strain D5 (Bar D5) against the well-known pathogen Fusarium oxysporum R1 (Fox R1) using different techniques in vitro and in vivo in saffron plants (Crocus sativus). The authors claim that this pathogen is a major problem for this crop and the disease caused by the pathogen, the corm rot, one of the main reasons to explain the progressive decline of its cultivation in India. Management of the disease is normally done with antifungal chemicals, with the subsequent negative effect in the environment. The authors propose that Bar D5 can be used in the future as a biocontrol agent to control the corm rot of saffron in replacement of antifungals.

Overall, this work describes extensively the inhibition of Fox R1 by Bar D5 and its protective effect over the plant, providing some novel insights with very simple and easy to understand experiments. The topic of the research proposed by the authors is interesting and their conclusions seem robust enough to merit publication. However, I have some concerns that, in the opinion of this reviewer, need to be addressed before publication; especially in some of the methods and in the way the data is presented.

Major comments:

Comment 1: In the methods section, in general, the methods need to be properly described. A reference is not enough to explain a technique. The paper would benefit from a more complete description, especially, in the preparation of the bioformulation of the strain Bar D5. This should be explained in detail or at least, should be briefly described along with a reference of the exhaustive preparation technique.

Response: As suggested, the detailed protocol of Bar D5 based bioformulation preparation has been added in the revised manuscript. The other missing information has also been added in the material and method section.

Comment 2: Also, in the q-PCR of used to quantify the pathogen load during infection, the article would benefit from a more detailed description regarding how the relative quantification was calculated. In addition, the authors chose as a reference gene a reference gene from the plant and not from the pathogen (the crocus 18S gene) is there any explanation? Seems, more logical to use a gene from the pathogen as a reference since the pathogen’s DNA is the one being quantified.

Response: The formula and method used for calculating the relative quantification of Fusarium oxysporum R1 in different samples has been added in detail in material and method section. The reference gene of saffron (18S rRNA gene) was used because the load of Fox R1 was quantified in-planta (in saffron corm). The infected tissue used for quantification of the load of pathogen consisted of DNA of both saffron and Fox R1. Since, the amount of the tissue taken for the pathogen load calculation was 100mg, the saffron internal primer was used as a control to measure increase in the fungal load as disease progressed, the results of q-PCR reflected that as fungal load increased as days progressed. Then the ct values obtained from control and test samples were used to calculate the relative quantification.

Comment 3: Fig.1 shows the inhibition of Fox R1 by Bar D5. The data presented is qualitative, and consists only of images of the plates with the pathogen in the presence or absence of Bar D5. The authors claim that “Bar D5 inhibits the growth of Fox R1 by 41%” (page 9 line 18). This needs to be supported by quantitative data and its corresponding statistical analysis. Quantification of the diameter of growth of the pathogen can be done in image J using the pictures of the plates and the resulting values can be used to calculate and plot the % of inhibition with its corresponding statistical analysis.

Response: As suggested, the diameter of growth of Fox R1 in both control and test plates were determined using ImageJ software and the values obtained were used for the calculation of % inhibition.

Comment 4: Authors claim that in their seal plate method to evaluate the production of volatile compounds by the bacterial colony, they observed a growth reduction of 25%. How is this percentage calculated? Based on what measurements? Quantitative data needs to be provided.

Response: % inhibition was calculated by measuring the diameter of growth of Fox R1 in both control and test plates. However, fresh calculations were done by subjecting the plates images into ImageJ software as suggested.

Comment 5: Fig. 2 and 3: The microscopy images presented have very poor quality and cannot be published in their current state. In addition, some of the images (Fig. 2 A and D, Fig. 3 F) look like they have been manually enlarged. Also, the images do not have a scale bar. New and better images are required if these data and its discussion are going to be kept in the final text of the article. In addition, please, provide the original images.

Response: Original, good quality images have been added in the revised manuscript.

Comment 6: Additionally, there is some confusion in the figure legends of Fig. 2 and 3, which need to be more descriptive and carefully revised. Legend in Fig. 2 mentions “complete loss of pigmentation”, which is something that cannot be observed in Fig. 2. There are also reference in Fig.2 to e f g or h which are not part of Fig. 2. The legend of Fig. 3 a refers to the length of hyphae, which are not part of that figure. These are just some examples.

Response: Thank you for pointing out this mistake, the figure legends have been corrected in the revised manuscript.

Comment 8: The bright field and fluorescence images in Fig. 4 should be presented separately along with the overlay.

Response: The bright field and fluorescence image corresponds of each overlay image has not been added as it is difficult to accommodate bright field and fluorescence images along with merged images in a single image. The main aim of adding these images are to differentiated between mock primed and Bar D5 primed corms and infected with Fox R1.

Comment 9: The data from the quantification of the Fox R1 load by CFU counts in the infected plants treated or untreated with Bar D5 is summarized on a table. A plot would be a much more informative way of presenting these data and it will show the statistical validation. This plot could go in the same figure as the one in Fig. 5.

Response: As suggested, the table has been converted into bar plot. However, the plot has not been merged with figure 7 as figure 7 is based on relative quantification of Fox R1 and figure 8 is based on absolute quantification of the Fox R1 in different treatments.

Comment 10: Concerning the characterization of the antagonism of Bar D5 against Fox R1 in planta, it would be very helpful, if feasible, to have also pictures of the infected corms treated or non-treated with Bar D5 to see differences in disease progression and/or severity. This data could also be accompanied by scanning electron microscopy images showing a detail of the fungal growth in treated plants compared to the untreated control.

Response: The picture of corms infected with Fox R1 in absence and presence of Bar D5 have been added in the revised manuscript.

Comment 11: Concerning the transcriptomic analysis of Fox R1 during infection in the presence of Bar D5, why did the authors choose 2 dpi as their condition? This needs to be better justified. Especially, considering that maximum inhibition is achieved at 5 dpi, according to the authors (page 10, line 52), which would also be an interesting time-point to study. Furthermore, what is the fold change threshold value used by the authors to consider a gene as differentially expressed?

Response: In the present study, 2dpi were selected for the study as it was midpoint from initial response at the zero hour of the infection to the 5dpi maximum response of the plant. Though, in future if we have resources we would want to conduct both the initial and extreme i.e 5dpi transcriptomics analysis studies. The genes with p<0.05 genes were considered significant and log2fold >0.5 in control vs test were considered differentially expressed genes.

Comment 12: In section 3.4, the authors comment on some of the differentially expressed genes that they found in the presence of Bar D5 during the infection. The reader would benefit from a small introduction in this section to make the story a bit more cohesive. Why are we talking about these genes specifically out of 3689 differentially expressed genes, according to the authors? Why is the alteration of their gene expression in the presence of Bar D5 important? Part of this is mentioned in the discussion section, but it is this reviewer opinion that the discussion section is too long, and the flow of the article would benefit from mentioning some of these points during the results section. Some parts of the discussion are also too exhaustive (for instance, page 15 lines 25-41). I believe that this section should be better summarized (or even shortened), in general.

Response: Thank you for the critical evaluation. In the present study, we have focused on the genes related to growth and pathogenicity only. These genes were identified by subjecting the total data to Pathogen-host interaction database. The alteration of these genes in the presence of Bar D5 is important because if these genes are getting down-regulated or getting repressed in presence of Bar D5 it means Bar D5 is an efficient biocontrol agent. The discussion part of the manuscript has been shortened.

Minor comments:

Comment: In the methods section, an accession number is provided for Bar D5 (KT228251) and the authors mention in page 4 line 7 a “draft genome sequence”. However, the accession number leads to a genbank page containing only the sequence of the 16S rRNA (https://www.ncbi.nlm.nih.gov/nuccore/KT228251).

Response: In the manuscript, the accession number of 16S rRNA gene has been given. The same has been mentioned in the revised manuscript.

Comment: In the discussion section, the authors mention that “The results of q-PCR revealed that the Bar D5 was better compared to carbendazim in controlling the growth of Fusarium oxysporum R1” (page 16 lines 16-19), but this is inaccurate, as carbendazim is more efficient at earlier time-points post infection.

Response: Yes, although carbendazim was better at earlier time point but in the latter stages biocontrol agent performed better. We have mentioned in the revised manuscript that Bar D5 is better in long run.

Comment: Authors mention several times throughout the text the expressions “was more” or “was less” (for example in page 10 line 56). I believe a better wording would be “was higher” or “was lower” (increased or decreased are also possible options in some parts of the text).

Response: Thank you for the critical evaluation, as suggested the word more has been replaced by higher.

**Reviews of manuscript FEMSMC-2023-006.r1**

**Reviewer 2**

**Recommendation: Minor Revision**

I genuinely appreciate the authors’ efforts in responding to my comments. They have addressed most of my concerns and they have submitted, in my opinion, an improved version of the manuscript. However, I believe that there are some minor details that still need to be addressed before finally accepting the manuscript for publication:

-I see that the authors have changed the title. I believe the previous title was better suited as the paper does not explore the mechanism of action of the biocontrol activity of Bar D5 against Fox R1, which in this case would be, in my opinion, to explore in detail which compounds produced by Bar D5 mediate this biocontrol effect and how is this achieved.

-Regarding the quantification of the inhibition of Fox R1 by Bar D5 (figure 1) and the quantification of the inhibition of Fox R1 due to the volatile compounds produced by Bar D5 from the sealed plate assay (figure 3), I appreciate the authors efforts in providing the values from the image based quantification. However, these quantitative data cannot be found anywhere in the manuscript, only in the description of the results (section 3.1 lines 3-7 and lines 22-37). Could the authors show these data in bar plots comparing the effect of Bar D5 to a control (for instance, Fox R1 alone) as part of figure 1 and 3 respectively?

-In addition, could the authors show in the different bar plots the individual data points of the replicates to quickly assess the dispersion range of the data?

-Regarding the new images provided for figure 2, I cannot help but wonder, what are the circles that appear over the three images? This is puzzling, especially when we compare these images with the ones shown in figure 4, that are the result of the same technique and have now much better quality.

-Concerning the transcriptomic analysis, the authors have mentioned in their response that they consider the genes as differentially expressed when they present a log2FC > 0.5. In principle, there is nothing wrong with that. However, the consensus when doing this type of analysis is to consider the genes that have, at least, a log2FC > 1, therefore, a fold change of 2, which means that a specific gene has an expression that is 2 fold higher in that specific condition compared to the control condition. I think that changing this threshold could help the authors in simplifying their analysis and conclusions and have a clearer picture.

-Discussion, lines 46-47: “This could be due to absence of BarD5 in control corms as they were mock primed”, what is this referring to? That line there is a bit confusing, as the line just before says “The effect of Bar D5 was evaluated against Fox R1 in saffron corms, and chlamydospores formation of Fox R1 were observed at 20 dpi, in the presence of Bar D5 indicating the fungistatic mode of action of Bar D5 (Fig. 6 p)”. What do the mock primed corms have to do here? Please, consider re-writing/deleting it to make sure that the whole paragraph is coherent.

**Reviewer 3**

**Recommendation: Accept**

Authors have done a careful revision and I don't have any additional requests.

**Author's Response to Decision Letter for (FEMSMC-2023-006.r1)**

I would like to thank you on behalf of the authors for considering our work in FEMS Microbes. The manuscript entitled “Effects of biocontrol Bacillus sp. strain D5 on the pathogenic Fusarium oxysporum R1 at microscopic and molecular level in Crocus sativus L. (saffron) corm” has been revised as per the suggestions of the reviewers.

The text, revised in the manuscript is highlighted in red.

Kindly find the point wise response to the comments /suggestions raised by the reviewers.

Associate Editor:

Both reviewers are pleased with the revisions and agree that most of the previous concerns and suggestions have been addressed. However, Reviewer 2 has pointed out a few areas in which the manuscript could be further improved. I agree with this assessment, and thus request that the authors make the following suggested minor revisions.

Comment 1: Consider changing title back to the original wording.

Response: As suggested, the title has been changed to original.

Comment 2: Include the quantification of the data shown in Figures 1 and 3 in bar charts or similar graphs, plotting the data in such a way that indicates the variation of the data. These charts should be presented as additional panels in each figure.

Response: Done as suggested

Comment 3: Revised the section of the discussion (lines 46-47) to make this point more clear.

Response: Done as suggested

Reviewer 2:

Comment 1: I genuinely appreciate the authors’ efforts in responding to my comments. They have addressed most of my concerns and they have submitted, in my opinion, an improved version of the manuscript. However, I believe that there are some minor details that still need to be addressed before finally accepting the manuscript for publication:-I see that the authors have changed the title. I believe the previous title was better suited as the paper does not explore the mechanism of action of the biocontrol activity of Bar D5 against Fox R1, which in this case would be, in my opinion, to explore in detail which compounds produced by Bar D5 mediate this biocontrol effect and how is this achieved.

Response: Thanks for the appreciation. As suggested, the title of the manuscript has changed to “Effects of biocontrol Bacillus sp. strain D5 on the pathogenic Fusarium oxysporum R1 at the microscopic and molecular level in Crocus sativus L. (saffron) corm”.

Comment 2: Regarding the quantification of the inhibition of Fox R1 by Bar D5 (figure 1) and the quantification of the inhibition of Fox R1 due to the volatile compounds produced by Bar D5 from the sealed plate assay (figure 3), I appreciate the authors efforts in providing the values from the image based quantification. However, these quantitative data cannot be found anywhere in the manuscript, only in the description of the results (section 3.1 lines 3-7 and lines 22-37). Could the authors show these data in bar plots comparing the effect of Bar D5 to a control (for instance, Fox R1 alone) as part of figure 1 and 3 respectively?-In addition, could the authors show in the different bar plots the individual data points of the replicates to quickly assess the dispersion range of the data?-

Response: Done as suggested

Comment 3: Regarding the new images provided for figure 2, I cannot help but wonder, what are the circles that appear over the three images? This is puzzling, especially when we compare these images with the ones shown in figure 4 that are the result of the same technique and have now much better quality.

Response: Thank you for pointing out this. The background of the image with circles is due to microscopic artifact and has been removed and replaced with good quality images. .

Comment 4: Concerning the transcriptomic analysis, the authors have mentioned in their response that they consider the genes as differentially expressed when they present a log2FC > 0.5. In principle, there is nothing wrong with that. However, the consensus when doing this type of analysis is to consider the genes that have, at least, a log2FC > 1, therefore, a fold change of 2, which means that a specific gene has an expression that is 2 fold higher in that specific condition compared to the control condition. I think that changing this threshold could help the authors in simplifying their analysis and conclusions and have a clearer picture.

Response: In the revised manuscript, we have mentioned about number of genes with p value <0.05 and log2fold>1. However, function of all the 60 genes has been mentioned.

Comment 5: Discussion, lines 46-47: “This could be due to absence of BarD5 in control corms as they were mock primed”, what is this referring to? That line there is a bit confusing, as the line just before says “The effect of Bar D5 was evaluated against Fox R1 in saffron corms, and chlamydospores formation of Fox R1 were observed at 20 dpi, in the presence of Bar D5 indicating the fungistatic mode of action of Bar D5 (Fig. 6 p)”. What do the mock primed corms have to do here? Please, consider re-writing/deleting it to make sure that the whole paragraph is coherent.

Response: Thank you for pointing out this. The line “This could be due to absence of BarD5 in control corms as they were mock primed” has been deleted from the revised manuscript.

Reviewer 3: Authors have done a careful revision and I don't have any additional requests.

Response: Thank you for appreciation.

**Reviews of manuscript FEMSMC-2023-006.r2**

**Reviewer 4: Barbara Kunkel, Washington University in Saint Louis (Associate Editor)**

**Recommendation: Accept**

the authors have made all the editorial changes requested
